# OpWise: Operons aid the identification of differentially expressed genes in bacterial microarray experiments

**DOI:** 10.1186/1471-2105-7-19

**Published:** 2006-01-13

**Authors:** Morgan N Price, Adam P Arkin, Eric J Alm

**Affiliations:** 1 Lawrence Berkeley Lab, 1 Cyclotron Road, Mailstop 977-152, Berkeley CA 94720, USA; 2 Virtual Institute of Microbial Stress and Survival; 3 Howard Hughes Medical Institute, Berkeley CA, USA; 4 University of California at Berkeley, Department of Bioengineering, Berkeley CA, USA

## Abstract

**Background:**

Differentially expressed genes are typically identified by analyzing the variation between replicate measurements. These procedures implicitly assume that there are no systematic errors in the data even though several sources of systematic error are known.

**Results:**

OpWise estimates the amount of systematic error in bacterial microarray data by assuming that genes in the same operon have matching expression patterns. OpWise then performs a Bayesian analysis of a linear model to estimate significance. In simulations, OpWise corrects for systematic error and is robust to deviations from its assumptions. In several bacterial data sets, significant amounts of systematic error are present, and replicate-based approaches overstate the confidence of the changers dramatically, while OpWise does not. Finally, OpWise can identify additional changers by assigning genes higher confidence if they are consistent with other genes in the same operon.

**Conclusion:**

Although microarray data can contain large amounts of systematic error, operons provide an external standard and allow for reasonable estimates of significance. OpWise is available at .

## Background

Microarray measurements of gene expression have become a popular tool for studying bacterial physiology, and hundreds of such studies are being conducted each year. Generally, these studies compare a treatment, either environmental or genetic, to a control condition. After obtaining raw hybridization intensities by scanning the slides or chips, the next steps are to normalize the data to remove experimental artifacts and then to identify differentially expressed genes.

To assess the reliability of the microarray measurements and to distinguish significant changers from other genes, statisticians have analyzed the variation between replicate experiments [1-8]. Implicitly, assessing significance by testing replication error assumes that replication captures all of the error in the data, and that there are no systematic biases. However, systematic errors have been observed due to many factors, including cross-hybridization, non-specific hybridization, dye incorporation bias, intensity-dependent effects, and spatial artifacts [1,9-11]. Although normalization methods correct for some of these, systematic bias will likely remain; for example, most normalization methods cannot account for cross-hybridization or non-specific hybridization. To determine if systematic errors do remain after normalization, additional information besides the replicates is required.

For bacterial microarray experiments, we use operons to assess the amount of systematic error in the data. Bacterial genes are often co-transcribed in multi-gene operons, and genes in the same operon should, in principle, have the same expression pattern. Although genes in the same operon are often expressed at different levels due to the varying stability of different segments of the mRNA, in steady-state situations, this will not affect the ratio in expression levels between conditions. Because most mRNA half-lives are short (under 10 minutes [12,13]), mRNA levels will be near steady state both in sustained growth (e.g., log phase) or within 20–30 minutes of a stress (e.g., heat) being applied. Thus, the steady state approximation should generally hold, and expression ratios should be consistent across an operon. Another reason why expression patterns can vary within an operon is that some operons have internal promoters or differential regulation of mRNA stability that can lead to differences in expression patterns [14]. In practice, however, genes known to be in the same operon usually have very similar expression patterns, and expression patterns can be used to predict operons [15].

We assume that genes in the same operon have identical expression patterns, and infer that differences between the expression patterns of genes in the same operon are due to errors, which may be systematic or not. This assumption is somewhat conservative, because any true differences in expression patterns between genes in the same operon will be mistaken for errors, leading to overestimation of the amount of systematic error and conservative assessments of significance. In practice, however, this effect appears to be slight. Because the operon structure of most genes has not been experimentally determined, we rely on operon predictions that are available for all prokaryotes [16], along with estimates of their reliability [17,18].

Given this assumption about operons, we wish to estimate the amount of systematic bias in the data. One simple test is to ask how often two genes that are in the same operon have the same direction of change. However, even if one of the genes is a confident changer, and even if the operon prediction is highly confident, the measurement for the other gene in the operon may be noisy. In this case, the second gene will often report a change in the opposite direction from the first gene because of variation between the replicate measurements, and not because of systematic bias. Thus, interpreting the external information from operons requires us to have a model of the replication error.

We extend linear models for microarray data with replicates [3,5,8] to include systematic errors, and present an empirical Bayes analysis of the overall amount of systematic error and of the significance of each gene. Because we have observed that even low-confidence changers show a significant amount of agreement with operons, we do not assume that a minority of genes are changers and that the rest of the genes do not change [5,8]. Instead, we will assume that all genes are changing, even if, for most of them, the magnitude of change is small and the direction of change cannot be determined with confidence. Consequently, rather than trying to distinguish the changers from the rest of the genes, we estimate for each gene the posterior distribution for the gene's fold-change given the data and the model. This can be summarized as a confidence interval, as the posterior probability that the gene's expression level went up (or down) in response to the treatment, or as the probability that the gene changed by 1.5-fold or more.

To test our method, we conducted simulations and also analyzed several experimental data sets. In simulations, the method correctly estimates the amount of systematic bias in the data and gives reasonable *p*-values even when some of the assumptions of the method are violated. On real data, we tested the agreement with operons of genes having varying levels of significance. For both two-color cDNA data and Affymetrix oligonucleotide data, our method finds significant amounts of systematic error and reports plausible *p*-values that show a gradual reduction in agreement with operons as significance decreases. In contrast, approaches based on replication error, including non-parametric approaches [4,6,7], often show low agreement with operons for confident changers (genes with > 99% probability of being true changers). Thus, methods that ignore systematic bias may be overstating significance dramatically.

We can also take advantage of operon structure to identify more changers. Intuitively, if two or three genes in the same operon all change in the same direction then they are unlikely to be false positives, but a changer that disagrees with the other genes in the same operon is suspect. Such reasoning is often used by biologists when examining microarray data. We derive a statistically sound "operon-wise" *p*-value, and show that these operon-wise *p*-values allow the identification of more changers at any specified level of significance than do single-gene *p*-values.

## Implementation

We present "OpWise," an empirical Bayes method for estimating the significance of the changes reported for each gene. The key elements of OpWise are (i) a linear error model that includes systematic errors, (ii) an approach for estimating the parameters of the error model (the hyperparameters), and, in particular, for inferring the amount of systematic error from the agreement within operons, (iii) a mathematical solution for the posterior distribution of a gene's change in expression given the data for the gene and the parametrized error model, and (iv) an extension to the method to take other genes in the same operon into account when estimating the significance of each gene.

To describe the expression of each gene, we use normalized expression ratios, as these should be consistent within each operon. In practice, we use log-ratios (base 2) rather than raw ratios. Also, instead of assuming that only a small fraction of genes are changing, we assume that every gene is changing (but only a small fraction of them might be measured with high confidence). Furthermore, we assume that there is some unknown amount of systematic error in the measurement for each gene, so that errors will remain no matter the number of replicates. Then, given the data for a gene *i*, we estimate the posterior distribution for the true log-ratio *μ*_*i*_. This distribution can be summarized with a confidence interval or with the probability *P*(*μ*_*i *_> 0) that a gene's expression level went up in the treatment condition. This probability will be near zero for highly confident down-changers, near one for highly confident up-changers, and near 0.5 for low-confidence measurements.

### A linear model with systematic errors

First consider a simple experimental design with direct comparisons, where the samples from the conditions being compared are hybridized to the same chip. Each gene *i *has an unknown true response *μ*_*i*_, systematic error *ε*_*i*_, and variance between replicates σi2
 MathType@MTEF@5@5@+=feaafiart1ev1aaatCvAUfKttLearuWrP9MDH5MBPbIqV92AaeXatLxBI9gBaebbnrfifHhDYfgasaacH8akY=wiFfYdH8Gipec8Eeeu0xXdbba9frFj0=OqFfea0dXdd9vqai=hGuQ8kuc9pgc9s8qqaq=dirpe0xb9q8qiLsFr0=vr0=vr0dc8meaabaqaciaacaGaaeqabaqabeGadaaakeaaiiGacqWFdpWCdaqhaaWcbaGaemyAaKgabaGaeGOmaidaaaaa@30F0@. The measurements x→i
 MathType@MTEF@5@5@+=feaafiart1ev1aaatCvAUfKttLearuWrP9MDH5MBPbIqV92AaeXatLxBI9gBaebbnrfifHhDYfgasaacH8akY=wiFfYdH8Gipec8Eeeu0xXdbba9frFj0=OqFfea0dXdd9vqai=hGuQ8kuc9pgc9s8qqaq=dirpe0xb9q8qiLsFr0=vr0=vr0dc8meaabaqaciaacaGaaeqabaqabeGadaaakeaacuWG4baEgaWcamaaBaaaleaacqWGPbqAaeqaaaaa@2FBE@ for gene *i *are assumed to be normally distributed around *μ*_*i *_+ *ε*_*i*_, and can be summarized by the observed mean *m*_*i *_= ∑_*j*_*x*_*ij*_/*n*_*i*_, where *n*_*i *_is the number of measurements for gene *i*, and the total squared deviance si2
 MathType@MTEF@5@5@+=feaafiart1ev1aaatCvAUfKttLearuWrP9MDH5MBPbIqV92AaeXatLxBI9gBaebbnrfifHhDYfgasaacH8akY=wiFfYdH8Gipec8Eeeu0xXdbba9frFj0=OqFfea0dXdd9vqai=hGuQ8kuc9pgc9s8qqaq=dirpe0xb9q8qiLsFr0=vr0=vr0dc8meaabaqaciaacaGaaeqabaqabeGadaaakeaacqWGZbWCdaqhaaWcbaGaemyAaKgabaGaeGOmaidaaaaa@3095@ = ∑_*j*_(*x*_*ij *_- *m*_*i*_)^2^, so that the likelihood of the data for each gene *i *is given by

f(x→i)=∏j=1nif(xij|μi,σi,εi)∝σi−niexp⁡(−∑j(xij−μi−εi)22σi2)=σi−niexp⁡(−ni(μi+εi−mi)2+si22σi2)     (1)
 MathType@MTEF@5@5@+=feaafiart1ev1aaatCvAUfKttLearuWrP9MDH5MBPbIqV92AaeXatLxBI9gBaebbnrfifHhDYfgasaacH8akY=wiFfYdH8Gipec8Eeeu0xXdbba9frFj0=OqFfea0dXdd9vqai=hGuQ8kuc9pgc9s8qqaq=dirpe0xb9q8qiLsFr0=vr0=vr0dc8meaabaqaciaacaGaaeqabaqabeGadaaakeaafaqabeWacaaabaGaemOzayMaeiikaGIafmiEaGNbaSaadaWgaaWcbaGaemyAaKgabeaakiabcMcaPiabg2da9maarahabaGaemOzaygaleaacqWGQbGAcqGH9aqpcqaIXaqmaeaacqWGUbGBdaWgaaadbaGaemyAaKgabeaaa0Gaey4dIunakmaabmaabaGaemiEaG3aaSbaaSqaaiabdMgaPjabdQgaQbqabaGcdaabbaqaaGGaciab=X7aTnaaBaaaleaacqWGPbqAaeqaaOGaeiilaWIae83Wdm3aaSbaaSqaaiabdMgaPbqabaGccqGGSaalaiaawEa7aiab=v7aLnaaBaaaleaacqWGPbqAaeqaaaGccaGLOaGaayzkaaaabaaabaGae8xhIuRae83Wdm3aa0baaSqaaiabdMgaPbqaaiab=jHiTiabd6gaUnaaBaaameaacqWGPbqAaeqaaaaakiGbcwgaLjabcIha4jabcchaWjabcIcaOiabgkHiTmaalaaabaWaaabeaeaacqGGOaakcqWG4baEdaWgaaWcbaGaemyAaKMaemOAaOgabeaakiabgkHiTiab=X7aTnaaBaaaleaacqWGPbqAaeqaaOGaeyOeI0Iae8xTdu2aaSbaaSqaaiabdMgaPbqabaGccqGGPaqkdaahaaWcbeqaaiabikdaYaaaaeaacqWGQbGAaeqaniabggHiLdaakeaacqaIYaGmcqWFdpWCdaqhaaWcbaGaemyAaKgabaGaeGOmaidaaaaakiabcMcaPaqaaaqaaiabg2da9iab=n8aZnaaDaaaleaacqWGPbqAaeaacqGHsislcqWGUbGBdaWgaaadbaGaemyAaKgabeaaaaGccyGGLbqzcqGG4baEcqGGWbaCcqGGOaakcqGHsisldaWcaaqaaiabd6gaUnaaBaaaleaacqWGPbqAaeqaaOGaeiikaGIae8hVd02aaSbaaSqaaiabdMgaPbqabaGccqGHRaWkcqWF1oqzdaWgaaWcbaGaemyAaKgabeaakiabgkHiTiabd2gaTnaaBaaaleaacqWGPbqAaeqaaOGaeiykaKYaaWbaaSqabeaacqaIYaGmaaGccqGHRaWkcqWGZbWCdaqhaaWcbaGaemyAaKgabaGaeGOmaidaaaGcbaGaeGOmaiJae83Wdm3aa0baaSqaaiabdMgaPbqaaiabikdaYaaaaaGccqGGPaqkaeaacaWLjaGaaCzcamaabmaabaGaeGymaedacaGLOaGaayzkaaaaaaaa@A627@

Another popular experimental design is to compare two types of samples separately to an external standard, such as genomic DNA or pooled mRNA samples. In these types of experiments, there are two sets of measured log levels for each gene, and the difference between them gives the log ratio. We refer to these log levels as x→1i
 MathType@MTEF@5@5@+=feaafiart1ev1aaatCvAUfKttLearuWrP9MDH5MBPbIqV92AaeXatLxBI9gBaebbnrfifHhDYfgasaacH8akY=wiFfYdH8Gipec8Eeeu0xXdbba9frFj0=OqFfea0dXdd9vqai=hGuQ8kuc9pgc9s8qqaq=dirpe0xb9q8qiLsFr0=vr0=vr0dc8meaabaqaciaacaGaaeqabaqabeGadaaakeaacuWG4baEgaWcamaaBaaaleaacqaIXaqmcqWGPbqAaeqaaaaa@30AE@ and x→2i
 MathType@MTEF@5@5@+=feaafiart1ev1aaatCvAUfKttLearuWrP9MDH5MBPbIqV92AaeXatLxBI9gBaebbnrfifHhDYfgasaacH8akY=wiFfYdH8Gipec8Eeeu0xXdbba9frFj0=OqFfea0dXdd9vqai=hGuQ8kuc9pgc9s8qqaq=dirpe0xb9q8qiLsFr0=vr0=vr0dc8meaabaqaciaacaGaaeqabaqabeGadaaakeaacuWG4baEgaWcamaaBaaaleaacqaIYaGmcqWGPbqAaeqaaaaa@30B0@, and summarize them with counts *n*_1*i *_and *n*_2*i*_, sample means *m*_1*i *_and *m*_2*i*_, and total squared deviances s1i2
 MathType@MTEF@5@5@+=feaafiart1ev1aaatCvAUfKttLearuWrP9MDH5MBPbIqV92AaeXatLxBI9gBaebbnrfifHhDYfgasaacH8akY=wiFfYdH8Gipec8Eeeu0xXdbba9frFj0=OqFfea0dXdd9vqai=hGuQ8kuc9pgc9s8qqaq=dirpe0xb9q8qiLsFr0=vr0=vr0dc8meaabaqaciaacaGaaeqabaqabeGadaaakeaacqWGZbWCdaqhaaWcbaGaeGymaeJaemyAaKgabaGaeGOmaidaaaaa@3185@ and s2i2
 MathType@MTEF@5@5@+=feaafiart1ev1aaatCvAUfKttLearuWrP9MDH5MBPbIqV92AaeXatLxBI9gBaebbnrfifHhDYfgasaacH8akY=wiFfYdH8Gipec8Eeeu0xXdbba9frFj0=OqFfea0dXdd9vqai=hGuQ8kuc9pgc9s8qqaq=dirpe0xb9q8qiLsFr0=vr0=vr0dc8meaabaqaciaacaGaaeqabaqabeGadaaakeaacqWGZbWCdaqhaaWcbaGaeGOmaiJaemyAaKgabaGaeGOmaidaaaaa@3187@. We assume that the true variance in measurements x→1i
 MathType@MTEF@5@5@+=feaafiart1ev1aaatCvAUfKttLearuWrP9MDH5MBPbIqV92AaeXatLxBI9gBaebbnrfifHhDYfgasaacH8akY=wiFfYdH8Gipec8Eeeu0xXdbba9frFj0=OqFfea0dXdd9vqai=hGuQ8kuc9pgc9s8qqaq=dirpe0xb9q8qiLsFr0=vr0=vr0dc8meaabaqaciaacaGaaeqabaqabeGadaaakeaacuWG4baEgaWcamaaBaaaleaacqaIXaqmcqWGPbqAaeqaaaaa@30AE@ and x→2i
 MathType@MTEF@5@5@+=feaafiart1ev1aaatCvAUfKttLearuWrP9MDH5MBPbIqV92AaeXatLxBI9gBaebbnrfifHhDYfgasaacH8akY=wiFfYdH8Gipec8Eeeu0xXdbba9frFj0=OqFfea0dXdd9vqai=hGuQ8kuc9pgc9s8qqaq=dirpe0xb9q8qiLsFr0=vr0=vr0dc8meaabaqaciaacaGaaeqabaqabeGadaaakeaacuWG4baEgaWcamaaBaaaleaacqaIYaGmcqWGPbqAaeqaaaaa@30B0@ is identical, and that the unknown systematic bias *ε*_*i *_affects the difference. We wish to estimate the distribution of *μ*_*i *_= *μ*_1*i *_- *μ*_2*i*_. Using the summary statistics *n*_*i *_= *n*_1*i *_+ *n*_2*i *_- 1, *N*_*i *_≡ (n1i−1
 MathType@MTEF@5@5@+=feaafiart1ev1aaatCvAUfKttLearuWrP9MDH5MBPbIqV92AaeXatLxBI9gBaebbnrfifHhDYfgasaacH8akY=wiFfYdH8Gipec8Eeeu0xXdbba9frFj0=OqFfea0dXdd9vqai=hGuQ8kuc9pgc9s8qqaq=dirpe0xb9q8qiLsFr0=vr0=vr0dc8meaabaqaciaacaGaaeqabaqabeGadaaakeaacqWGUbGBdaqhaaWcbaGaeGymaeJaemyAaKgabaGaeyOeI0IaeGymaedaaaaa@3266@ + n2i−1
 MathType@MTEF@5@5@+=feaafiart1ev1aaatCvAUfKttLearuWrP9MDH5MBPbIqV92AaeXatLxBI9gBaebbnrfifHhDYfgasaacH8akY=wiFfYdH8Gipec8Eeeu0xXdbba9frFj0=OqFfea0dXdd9vqai=hGuQ8kuc9pgc9s8qqaq=dirpe0xb9q8qiLsFr0=vr0=vr0dc8meaabaqaciaacaGaaeqabaqabeGadaaakeaacqWGUbGBdaqhaaWcbaGaeGOmaiJaemyAaKgabaGaeyOeI0IaeGymaedaaaaa@3268@)^-1^, *m*_*i *_≡ *m*_1*i *_- *m*_2*i*_, and si2
 MathType@MTEF@5@5@+=feaafiart1ev1aaatCvAUfKttLearuWrP9MDH5MBPbIqV92AaeXatLxBI9gBaebbnrfifHhDYfgasaacH8akY=wiFfYdH8Gipec8Eeeu0xXdbba9frFj0=OqFfea0dXdd9vqai=hGuQ8kuc9pgc9s8qqaq=dirpe0xb9q8qiLsFr0=vr0=vr0dc8meaabaqaciaacaGaaeqabaqabeGadaaakeaacqWGZbWCdaqhaaWcbaGaemyAaKgabaGaeGOmaidaaaaa@3095@ ≡ s1i2
 MathType@MTEF@5@5@+=feaafiart1ev1aaatCvAUfKttLearuWrP9MDH5MBPbIqV92AaeXatLxBI9gBaebbnrfifHhDYfgasaacH8akY=wiFfYdH8Gipec8Eeeu0xXdbba9frFj0=OqFfea0dXdd9vqai=hGuQ8kuc9pgc9s8qqaq=dirpe0xb9q8qiLsFr0=vr0=vr0dc8meaabaqaciaacaGaaeqabaqabeGadaaakeaacqWGZbWCdaqhaaWcbaGaeGymaeJaemyAaKgabaGaeGOmaidaaaaa@3185@ + s2i2
 MathType@MTEF@5@5@+=feaafiart1ev1aaatCvAUfKttLearuWrP9MDH5MBPbIqV92AaeXatLxBI9gBaebbnrfifHhDYfgasaacH8akY=wiFfYdH8Gipec8Eeeu0xXdbba9frFj0=OqFfea0dXdd9vqai=hGuQ8kuc9pgc9s8qqaq=dirpe0xb9q8qiLsFr0=vr0=vr0dc8meaabaqaciaacaGaaeqabaqabeGadaaakeaacqWGZbWCdaqhaaWcbaGaeGOmaiJaemyAaKgabaGaeGOmaidaaaaa@3187@, the likelihood is

f(mi,si2|μi,σi,εi)∝σi−niexp⁡(−Ni(μi+εi−mi)2+si22σi2)     (2)
 MathType@MTEF@5@5@+=feaafiart1ev1aaatCvAUfKttLearuWrP9MDH5MBPbIqV92AaeXatLxBI9gBaebbnrfifHhDYfgasaacH8akY=wiFfYdH8Gipec8Eeeu0xXdbba9frFj0=OqFfea0dXdd9vqai=hGuQ8kuc9pgc9s8qqaq=dirpe0xb9q8qiLsFr0=vr0=vr0dc8meaabaqaciaacaGaaeqabaqabeGadaaakeaacqWGMbGzdaqadaqaaiabd2gaTnaaBaaaleaacqWGPbqAaeqaaOGaeiilaWIaem4Cam3aa0baaSqaaiabdMgaPbqaaiabikdaYaaakmaaeeaabaacciGae8hVd02aaSbaaSqaaiabdMgaPbqabaGccqGGSaalcqWFdpWCdaWgaaWcbaGaemyAaKgabeaakiabcYcaSiab=v7aLnaaBaaaleaacqWGPbqAaeqaaaGccaGLhWoaaiaawIcacaGLPaaacqWFDisTcqWFdpWCdaqhaaWcbaGaemyAaKgabaGae8NeI0IaemOBa42aaSbaaWqaaiabdMgaPbqabaaaaOGagiyzauMaeiiEaGNaeiiCaaNaeiikaGIaeyOeI0YaaSaaaeaacqWGobGtdaWgaaWcbaGaemyAaKgabeaakiabcIcaOiab=X7aTnaaBaaaleaacqWGPbqAaeqaaOGaey4kaSIae8xTdu2aaSbaaSqaaiabdMgaPbqabaGccqGHsislcqWGTbqBdaWgaaWcbaGaemyAaKgabeaakiabcMcaPmaaCaaaleqabaGaeGOmaidaaOGaey4kaSIaem4Cam3aa0baaSqaaiabdMgaPbqaaiabikdaYaaaaOqaaiabikdaYiab=n8aZnaaDaaaleaacqWGPbqAaeaacqaIYaGmaaaaaOGaeiykaKIaaCzcaiaaxMaadaqadaqaaiabikdaYaGaayjkaiaawMcaaaaa@7285@

which is the same form as the direct comparison case except that *N*_*i *_has replaced *n*_*i *_in the exponential.

In either case, we use the conjugate prior to make the problem analytically tractable (as in [5,8]). We first assume that the distribution of *θ*_*i *_≡ 1/σi2
 MathType@MTEF@5@5@+=feaafiart1ev1aaatCvAUfKttLearuWrP9MDH5MBPbIqV92AaeXatLxBI9gBaebbnrfifHhDYfgasaacH8akY=wiFfYdH8Gipec8Eeeu0xXdbba9frFj0=OqFfea0dXdd9vqai=hGuQ8kuc9pgc9s8qqaq=dirpe0xb9q8qiLsFr0=vr0=vr0dc8meaabaqaciaacaGaaeqabaqabeGadaaakeaaiiGacqWFdpWCdaqhaaWcbaGaemyAaKgabaGaeGOmaidaaaaa@30F0@ follows a chi-squared distribution (Eq. 3). Given σi2
 MathType@MTEF@5@5@+=feaafiart1ev1aaatCvAUfKttLearuWrP9MDH5MBPbIqV92AaeXatLxBI9gBaebbnrfifHhDYfgasaacH8akY=wiFfYdH8Gipec8Eeeu0xXdbba9frFj0=OqFfea0dXdd9vqai=hGuQ8kuc9pgc9s8qqaq=dirpe0xb9q8qiLsFr0=vr0=vr0dc8meaabaqaciaacaGaaeqabaqabeGadaaakeaaiiGacqWFdpWCdaqhaaWcbaGaemyAaKgabaGaeGOmaidaaaaa@30F0@ for a gene, we then assume that the true mean *μ*_*i*_, is normally distributed with variance proportionate to σi2
 MathType@MTEF@5@5@+=feaafiart1ev1aaatCvAUfKttLearuWrP9MDH5MBPbIqV92AaeXatLxBI9gBaebbnrfifHhDYfgasaacH8akY=wiFfYdH8Gipec8Eeeu0xXdbba9frFj0=OqFfea0dXdd9vqai=hGuQ8kuc9pgc9s8qqaq=dirpe0xb9q8qiLsFr0=vr0=vr0dc8meaabaqaciaacaGaaeqabaqabeGadaaakeaaiiGacqWFdpWCdaqhaaWcbaGaemyAaKgabaGaeGOmaidaaaaa@30F0@. This assumption fits our data better than the alternative assumption of a fixed variance of *μ*_*i *_across all genes (see Results), and previous work also used this proportionality [8]. We use the same proportionality for the systematic error *ε*_*i*_. Hence, our prior is:

θi≡1/σi2θi⋅α~χ2(ν+1)f(θi)=θiν−12e−αθi2(α2)ν+12Γ(ν+12)μi~N(0,1θiβ)εi~N(0,1θiγ)     (3)
 MathType@MTEF@5@5@+=feaafiart1ev1aaatCvAUfKttLearuWrP9MDH5MBPbIqV92AaeXatLxBI9gBaebbnrfifHhDYfgasaacH8akY=wiFfYdH8Gipec8Eeeu0xXdbba9frFj0=OqFfea0dXdd9vqai=hGuQ8kuc9pgc9s8qqaq=dirpe0xb9q8qiLsFr0=vr0=vr0dc8meaabaqaciaacaGaaeqabaqabeGadaaakeaafaqabeqbcaaaaeaaiiGacqWF4oqCdaWgaaWcbaGaemyAaKgabeaakiabggMi6kabigdaXiabc+caViab=n8aZnaaDaaaleaacqWGPbqAaeaacqaIYaGmaaaakeaaaeaacqWF4oqCdaWgaaWcbaGaemyAaKgabeaakiabgwSixlab=f7aHjabc6ha+jab=D8aJnaaCaaaleqabaGaeGOmaidaaOGaeiikaGIae8xVd4Maey4kaSIaeGymaeJaeiykaKcabaaabaGaemOzayMaeiikaGIae8hUde3aaSbaaSqaaiabdMgaPbqabaGccqGGPaqkcqGH9aqpdaWcaaqaaiab=H7aXnaaDaaaleaacqWGPbqAaeaadaWcbaadbaGae8xVd4MaeyOeI0IaeGymaedabaGaeGOmaidaaaaakiabdwgaLnaaCaaaleqabaGaeyOeI0YaaSqaaWqaaiab=f7aHjab=H7aXnaaBaaabaGaemyAaKgabeaaaeaacqaIYaGmaaaaaOWaaeWaaeaadaWcbaWcbaGae8xSdegabaGaeGOmaidaaaGccaGLOaGaayzkaaWaaWbaaSqabeaadaWcbaadbaGae8xVd4Maey4kaSIaeGymaedabaGaeGOmaidaaaaaaOqaaiabfo5ahnaabmaabaWaaSqaaSqaaiab=17aUjabgUcaRiabigdaXaqaaiabikdaYaaaaOGaayjkaiaawMcaaaaaaeaaaeaacqWF8oqBdaWgaaWcbaGaemyAaKgabeaakiabc6ha+jabd6eaojabcIcaOiabicdaWiabcYcaSmaalaaabaGaeGymaedabaGae8hUde3aaSbaaSqaaiabdMgaPbqabaGccqWFYoGyaaGaeiykaKcabaaabaGae8xTdu2aaSbaaSqaaiabdMgaPbqabaGccqGG+bGFcqWGobGtcqGGOaakcqaIWaamcqGGSaaldaWcaaqaaiabigdaXaqaaiab=H7aXnaaBaaaleaacqWGPbqAaeqaaOGae83SdCgaaiabcMcaPaqaaiaaxMaacaWLjaWaaeWaaeaacqaIZaWmaiaawIcacaGLPaaaaaaaaa@931C@

with hyperparameters *α*, *ν*, *β*, and *γ*. 1/*α *is the scale of the chi-squared, *ν *+ 1 is its degrees of freedom, 1/*β *determines the amount of true changes in expression, and 1/*γ *determines the amount of systematic error.

We assume that the true means for the genes are independent, except that genes in the same operon have the same *θ*_*i *_and *μ*_*i *_(but independent bias *ε*_*i*_). Genes in the same operon are co-regulated, so *μ*_*i *_should be similar. The assumption that *θ*_*i *_is identical is required because in our model *μ*_*i *_depends on *θ*_*i *_the effectiveness of this assumption will be tested in the Results. Because operon predictions are only 80–90% accurate, we use a method that estimates the probability *P*(*Operon*_*ij*_) that two adjacent genes are co-transcribed [16], and treat the actual state of each potential operon pair as an unknown random variable. For example, the prediction method might estimate that two genes have a 90% probability of being in the same operon; in our model, we use this estimate as the true probability. We use only the likely operon pairs (those with *P*(*Operon*_*ij*_) ≥ 0.5).

### Solving a simplified model

We first describe how to solve a simplified model with systematic errors removed, so that *γ *= ∞ and thus all *ε*_*i *_= 0. We need to estimate the hyperparameters from the data, so that we have a fully specified prior distribution, and then we need to infer the posterior distribution of the log-fold-change *μ*_*i *_for each gene.

#### Estimating the hyperparameters

In this simplified model, we need to estimate the prior distribution for *θ*_*i *_(or σi2
 MathType@MTEF@5@5@+=feaafiart1ev1aaatCvAUfKttLearuWrP9MDH5MBPbIqV92AaeXatLxBI9gBaebbnrfifHhDYfgasaacH8akY=wiFfYdH8Gipec8Eeeu0xXdbba9frFj0=OqFfea0dXdd9vqai=hGuQ8kuc9pgc9s8qqaq=dirpe0xb9q8qiLsFr0=vr0=vr0dc8meaabaqaciaacaGaaeqabaqabeGadaaakeaaiiGacqWFdpWCdaqhaaWcbaGaemyAaKgabaGaeGOmaidaaaaa@30F0@), which is determined by the scale *γ *and degrees of freedom *ν*, and then the scale of variation for the true log-ratio *μ*_*i *_given the variance σi2
 MathType@MTEF@5@5@+=feaafiart1ev1aaatCvAUfKttLearuWrP9MDH5MBPbIqV92AaeXatLxBI9gBaebbnrfifHhDYfgasaacH8akY=wiFfYdH8Gipec8Eeeu0xXdbba9frFj0=OqFfea0dXdd9vqai=hGuQ8kuc9pgc9s8qqaq=dirpe0xb9q8qiLsFr0=vr0=vr0dc8meaabaqaciaacaGaaeqabaqabeGadaaakeaaiiGacqWFdpWCdaqhaaWcbaGaemyAaKgabaGaeGOmaidaaaaa@30F0@, which is given by 1/*β*. Although we assume that *μ*_*i *_is normally distributed for all genes, instead of being allowed to vary for a minority of genes, the variation between replicates in our model is the same as in [8]. As discussed by [8], logs si2
 MathType@MTEF@5@5@+=feaafiart1ev1aaatCvAUfKttLearuWrP9MDH5MBPbIqV92AaeXatLxBI9gBaebbnrfifHhDYfgasaacH8akY=wiFfYdH8Gipec8Eeeu0xXdbba9frFj0=OqFfea0dXdd9vqai=hGuQ8kuc9pgc9s8qqaq=dirpe0xb9q8qiLsFr0=vr0=vr0dc8meaabaqaciaacaGaaeqabaqabeGadaaakeaacqWGZbWCdaqhaaWcbaGaemyAaKgabaGaeGOmaidaaaaa@3095@ (the log of the squared deviances) is approximately normally distributed, and its mean and variance can be written analytically. By fitting the hyperparameters *α *and *ν *to the observed mean and variance of log si2
 MathType@MTEF@5@5@+=feaafiart1ev1aaatCvAUfKttLearuWrP9MDH5MBPbIqV92AaeXatLxBI9gBaebbnrfifHhDYfgasaacH8akY=wiFfYdH8Gipec8Eeeu0xXdbba9frFj0=OqFfea0dXdd9vqai=hGuQ8kuc9pgc9s8qqaq=dirpe0xb9q8qiLsFr0=vr0=vr0dc8meaabaqaciaacaGaaeqabaqabeGadaaakeaacqWGZbWCdaqhaaWcbaGaemyAaKgabaGaeGOmaidaaaaa@3095@, [8] derived the following estimator:

ei≡log⁡si2−ψ(ni−12)+log⁡(ni−12)ψ'(ν+12)= mean{(ei−e¯)2.NgenesNgenes−1−ψ'(ni−12)}αν+1=exp⁡{e¯+ψ(ν+12)−log⁡(ν+12)}     (4)
 MathType@MTEF@5@5@+=feaafiart1ev1aaatCvAUfKttLearuWrP9MDH5MBPbIqV92AaeXatLxBI9gBaebbnrfifHhDYfgasaacH8akY=wiFfYdH8Gipec8Eeeu0xXdbba9frFj0=OqFfea0dXdd9vqai=hGuQ8kuc9pgc9s8qqaq=dirpe0xb9q8qiLsFr0=vr0=vr0dc8meaabaqaciaacaGaaeqabaqabeGadaaakeaafaqabeWacaaabaGaemyzau2aaSbaaSqaaiabdMgaPbqabaGccqGHHjIUcyGGSbaBcqGGVbWBcqGGNbWzcqWGZbWCdaqhaaWcbaGaemyAaKgabaGaeGOmaidaaOGaeyOeI0ccciGae8hYdKNaeiikaGYaaSaaaeaacqWGUbGBdaWgaaWcbaGaemyAaKgabeaakiabgkHiTiabigdaXaqaaiabikdaYaaacqGGPaqkcqGHRaWkcyGGSbaBcqGGVbWBcqGGNbWzcqGGOaakdaWcaaqaaiabd6gaUnaaBaaaleaacqWGPbqAaeqaaOGaeyOeI0IaeGymaedabaGaeGOmaidaaiabcMcaPaqaaaqaaiab=H8a5jabcEcaNiabcIcaOmaalaaabaGae8xVd4Maey4kaSIaeGymaedabaGaeGOmaidaaiabcMcaPiabg2da9iabbccaGiabb2gaTjabbwgaLjabbggaHjabb6gaUjabbUha7jabbIcaOiabdwgaLnaaBaaaleaacqWGPbqAaeqaaOGaeyOeI0IafmyzauMbaebacqGGPaqkdaahaaWcbeqaaiabikdaYaaakiabc6caUmaalaaabaGaemOta40aaSbaaSqaaiabdEgaNjabdwgaLjabd6gaUjabdwgaLjabdohaZbqabaaakeaacqWGobGtdaWgaaWcbaGaem4zaCMaemyzauMaemOBa4MaemyzauMaem4CamhabeaakiabgkHiTiabigdaXaaacqWFsislcqWFipqEieaacqGFNaWjcqGFOaakdaWcaaqaaiabd6gaUnaaBaaaleaacqWGPbqAaeqaaOGaeyOeI0IaeGymaedabaGaeGOmaidaaiabcMcaPiabc2ha9bqaaaqaamaalaaabaGae8xSdegabaGae8xVd4Maey4kaSIaeGymaedaaiabg2da9iGbcwgaLjabcIha4jabcchaWjabcUha7jqbdwgaLzaaraGaey4kaSIae8hYdKNaeiikaGYaaSaaaeaacqWF9oGBcqGHRaWkcqaIXaqmaeaacqaIYaGmaaGaeiykaKIaeyOeI0IagiiBaWMaei4Ba8Maei4zaCMaeiikaGYaaSaaaeaacqWF9oGBcqGHRaWkcqaIXaqmaeaacqaIYaGmaaGaeiykaKIaeiyFa0habaGaaCzcaiaaxMaadaqadaqaaiabisda0aGaayjkaiaawMcaaaaaaaa@B05A@

where *ψ*() is the digamma function, *ψ'*() is the trigamma function, and e¯
 MathType@MTEF@5@5@+=feaafiart1ev1aaatCvAUfKttLearuWrP9MDH5MBPbIqV92AaeXatLxBI9gBaebbnrfifHhDYfgasaacH8akY=wiFfYdH8Gipec8Eeeu0xXdbba9frFj0=OqFfea0dXdd9vqai=hGuQ8kuc9pgc9s8qqaq=dirpe0xb9q8qiLsFr0=vr0=vr0dc8meaabaqaciaacaGaaeqabaqabeGadaaakeaacuWGLbqzgaqeaaaa@2E17@ is the mean of the *e*_*i*_. *ν *can be obtained by inverting the trigamma function, which can be preformed numerically by Newton iteration [8]. This leads to an estimate for *α *as well, and specifies the prior distribution of the true variances σi2
 MathType@MTEF@5@5@+=feaafiart1ev1aaatCvAUfKttLearuWrP9MDH5MBPbIqV92AaeXatLxBI9gBaebbnrfifHhDYfgasaacH8akY=wiFfYdH8Gipec8Eeeu0xXdbba9frFj0=OqFfea0dXdd9vqai=hGuQ8kuc9pgc9s8qqaq=dirpe0xb9q8qiLsFr0=vr0=vr0dc8meaabaqaciaacaGaaeqabaqabeGadaaakeaaiiGacqWFdpWCdaqhaaWcbaGaemyAaKgabaGaeGOmaidaaaaa@30F0@ for each gene (Eq. 3).

We then find the maximum likelihood estimate of *β*, which describes the prior distribution of the true means μi2
 MathType@MTEF@5@5@+=feaafiart1ev1aaatCvAUfKttLearuWrP9MDH5MBPbIqV92AaeXatLxBI9gBaebbnrfifHhDYfgasaacH8akY=wiFfYdH8Gipec8Eeeu0xXdbba9frFj0=OqFfea0dXdd9vqai=hGuQ8kuc9pgc9s8qqaq=dirpe0xb9q8qiLsFr0=vr0=vr0dc8meaabaqaciaacaGaaeqabaqabeGadaaakeaaiiGacqWF8oqBdaqhaaWcbaGaemyAaKgabaGaeGOmaidaaaaa@30E3@ for each gene (Eq. 3). The likelihood of the data is

f(m→,s2→)=∏if(mi,si2)=∏i∫0∞dθif(θi)∫−∞∞dμif(μi|θi)f(mi,si2|μi,θi)∝∏iββ+Ni⋅(α+si2+mi2⋅Ni⋅ββ+Ni)−ν+ni+12     (5)
 MathType@MTEF@5@5@+=feaafiart1ev1aaatCvAUfKttLearuWrP9MDH5MBPbIqV92AaeXatLxBI9gBaebbnrfifHhDYfgasaacH8akY=wiFfYdH8Gipec8Eeeu0xXdbba9frFj0=OqFfea0dXdd9vqai=hGuQ8kuc9pgc9s8qqaq=dirpe0xb9q8qiLsFr0=vr0=vr0dc8meaabaqaciaacaGaaeqabaqabeGadaaakeaafaqabeWacaaabaGaemOzayMaeiikaGIafmyBa0MbaSaacqGGSaalcqWGZbWCdaahaaWcbeqaaiqbikdaYyaalaaaaOGaeiykaKIaeyypa0ZaaebuaeaacqWGMbGzcqGGOaakcqWGTbqBdaWgaaWcbaGaemyAaKgabeaakiabcYcaSiabdohaZnaaDaaaleaacqWGPbqAaeaacqaIYaGmaaGccqGGPaqkaSqaaiabdMgaPbqab0Gaey4dIunaaOqaaaqaaiabg2da9maarafabaWaa8qmaeaacqWGKbaziiGacqWF4oqCdaWgaaWcbaGaemyAaKgabeaakiabdAgaMjabcIcaOiab=H7aXnaaBaaaleaacqWGPbqAaeqaaOGaeiykaKcaleaacqaIWaamaeaacqWFEisPa0Gaey4kIipaaSqaaiabdMgaPbqab0Gaey4dIunakmaapedabaGaemizaqMae8hVd02aaSbaaSqaaiabdMgaPbqabaGccqWGMbGzcqGGOaakcqWF8oqBdaWgaaWcbaGaemyAaKgabeaakmaaeeaabaGae8hUde3aaSbaaSqaaiabdMgaPbqabaaakiaawEa7aiabcMcaPaWcbaGae8NeI0Iae8NhIukabaGae8NhIukaniabgUIiYdGccqWGMbGzcqGGOaakcqWGTbqBdaWgaaWcbaGaemyAaKgabeaakiabcYcaSiabdohaZnaaDaaaleaacqWGPbqAaeaacqaIYaGmaaGcdaabbaqaaiab=X7aTnaaBaaaleaacqWGPbqAaeqaaOGaeiilaWIae8hUde3aaSbaaSqaaiabdMgaPbqabaGccqGGPaqkaiaawEa7aaqaaaqaaiab=1Hi1oaarafabaWaaOaaaeaadaWcaaqaaiab=j7aIbqaaiab=j7aIjabgUcaRiabd6eaonaaBaaaleaacqWGPbqAaeqaaaaaaeqaaaqaaiabdMgaPbqab0Gaey4dIunakiabgwSixpaabmaabaGae8xSdeMaey4kaSIaem4Cam3aa0baaSqaaiabdMgaPbqaaiabikdaYaaakiabgUcaRiabd2gaTnaaDaaaleaacqWGPbqAaeaacqaIYaGmaaGccqGHflY1daWcaaqaaiabd6eaonaaBaaaleaacqWGPbqAaeqaaOGaeyyXICTae8NSdigabaGae8NSdiMaey4kaSIaemOta40aaSbaaSqaaiabdMgaPbqabaaaaaGccaGLOaGaayzkaaWaaWbaaSqabeaacqGHsisldaWcbaadbaGae8xVd4Maey4kaSIaemOBa42aaSbaaeaacqWGPbqAaeqaaiabgUcaRiabigdaXaqaaiabikdaYaaaaaaakeaacaWLjaGaaCzcamaabmaabaGaeGynaudacaGLOaGaayzkaaaaaaaa@B79B@

where for direct comparison experiments, *N*_*i *_≡ *n*_*i*_. This equation can be viewed as a product of *t*-distributions for the posterior probabilities of each gene's measurements. We choose *β *to maximize the (logarithm of) this likelihood, using a Newton iteration method (*nlm *in the R statistics package: ).

#### Significance of individual genes

Given estimates for the hyperparameters and the observed mean *m*_*i *_and total squared deviance si2
 MathType@MTEF@5@5@+=feaafiart1ev1aaatCvAUfKttLearuWrP9MDH5MBPbIqV92AaeXatLxBI9gBaebbnrfifHhDYfgasaacH8akY=wiFfYdH8Gipec8Eeeu0xXdbba9frFj0=OqFfea0dXdd9vqai=hGuQ8kuc9pgc9s8qqaq=dirpe0xb9q8qiLsFr0=vr0=vr0dc8meaabaqaciaacaGaaeqabaqabeGadaaakeaacqWGZbWCdaqhaaWcbaGaemyAaKgabaGaeGOmaidaaaaa@3095@ for a gene *i*, the posterior probability distribution for *μ*_*i *_is given by

f(μi|mi,si2)∝∫0∞f(θi)f(μi|θi)f(mi,si2|θi,μi)dθi∝(α+βμi2+Ni(μi−mi)2+si2)−ν+ni2−1     (6)
 MathType@MTEF@5@5@+=feaafiart1ev1aaatCvAUfKttLearuWrP9MDH5MBPbIqV92AaeXatLxBI9gBaebbnrfifHhDYfgasaacH8akY=wiFfYdH8Gipec8Eeeu0xXdbba9frFj0=OqFfea0dXdd9vqai=hGuQ8kuc9pgc9s8qqaq=dirpe0xb9q8qiLsFr0=vr0=vr0dc8meaabaqaciaacaGaaeqabaqabeGadaaakeaafaqabeGacaaabaGaemOzay2aaeWaaeaaiiGacqWF8oqBdaWgaaWcbaGaemyAaKgabeaakmaaeeaabaGaemyBa02aaSbaaSqaaiabdMgaPbqabaGccqGGSaalcqWGZbWCdaqhaaWcbaGaemyAaKgabaGaeGOmaidaaaGccaGLhWoaaiaawIcacaGLPaaacqWFDisTdaWdXaqaaiabdAgaMnaabmaabaGae8hUde3aaSbaaSqaaiabdMgaPbqabaaakiaawIcacaGLPaaacqWGMbGzdaqadaqaaiab=X7aTnaaBaaaleaacqWGPbqAaeqaaOWaaqqaaeaacqWF4oqCdaWgaaWcbaGaemyAaKgabeaaaOGaay5bSdaacaGLOaGaayzkaaaaleaacqaIWaamaeaacqWFEisPa0Gaey4kIipakiabdAgaMnaabmaabaGaemyBa02aaSbaaSqaaiabdMgaPbqabaGccqGGSaalcqWGZbWCdaqhaaWcbaGaemyAaKgabaGaeGOmaidaaOWaaqqaaeaacqWF4oqCdaWgaaWcbaGaemyAaKgabeaakiabcYcaSiab=X7aTnaaBaaaleaacqWGPbqAaeqaaaGccaGLhWoaaiaawIcacaGLPaaacqWGKbazcqWF4oqCdaWgaaWcbaGaemyAaKgabeaaaOqaaaqaaiab=1Hi1oaabmaabaGae8xSdeMaey4kaSIae8NSdiMae8hVd02aa0baaSqaaiabdMgaPbqaaiabikdaYaaakiabgUcaRiabd6eaonaaBaaaleaacqWGPbqAaeqaaOWaaeWaaeaacqWF8oqBdaWgaaWcbaGaemyAaKgabeaakiabgkHiTiabd2gaTnaaBaaaleaacqWGPbqAaeqaaaGccaGLOaGaayzkaaWaaWbaaSqabeaacqaIYaGmaaGccqGHRaWkcqWGZbWCdaqhaaWcbaGaemyAaKgabaGaeGOmaidaaaGccaGLOaGaayzkaaWaaWbaaSqabeaacqGHsisldaWcbaadbaGae8xVd4Maey4kaSIaemOBa42aaSbaaeaacqWGPbqAaeqaaaqaaiabikdaYaaaliabgkHiTiabigdaXaaaaOqaaiaaxMaacaWLjaWaaeWaaeaacqaI2aGnaiaawIcacaGLPaaaaaaaaa@9676@

which is a *t *distribution with

mean=mi⋅Niβ+Nivariance=α+si2+mi2⋅Ni⋅ββ+Ni(β+Ni)⋅(ν+ni+1)d.f. = ν+ni+1     (7)
 MathType@MTEF@5@5@+=feaafiart1ev1aaatCvAUfKttLearuWrP9MDH5MBPbIqV92AaeXatLxBI9gBaebbnrfifHhDYfgasaacH8akY=wiFfYdH8Gipec8Eeeu0xXdbba9frFj0=OqFfea0dXdd9vqai=hGuQ8kuc9pgc9s8qqaq=dirpe0xb9q8qiLsFr0=vr0=vr0dc8meaabaqaciaacaGaaeqabaqabeGadaaakeaafaqabeWacaaabaGaeeyBa0MaeeyzauMaeeyyaeMaeeOBa4Maeyypa0ZaaSaaaeaacqqGTbqBdaWgaaWcbaGaeeyAaKgabeaakiabgwSixlabb6eaonaaBaaaleaacqqGPbqAaeqaaaGcbaacciGae8NSdiMaey4kaSIaeeOta40aaSbaaSqaaiabbMgaPbqabaaaaaGcbaaabaGaeeODayNaeeyyaeMaeeOCaiNaeeyAaKMaeeyyaeMaeeOBa4Maee4yamMaeeyzauMaeyypa0ZaaSaaaeaacqWFXoqycqGHRaWkieaacqGFZbWCdaqhaaWcbaGaeeyAaKgabaGaeGOmaidaaOGaey4kaSIaeeyBa02aa0baaSqaaiabbMgaPbqaaiabbkdaYaaakiabgwSixlabb6eaonaaBaaaleaacqqGPbqAaeqaaOGaeyyXIC9aaSqaaSqaaiab=j7aIbqaaiab=j7aIjabgUcaRiabb6eaonaaBaaameaacqqGPbqAaeqaaaaaaOqaaiabbIcaOiab=j7aIjabgUcaRiabb6eaonaaBaaaleaacqqGPbqAaeqaaOGaeiykaKIaeyyXICTaeiikaGIae8xVd4Maey4kaSIae4NBa42aaSbaaSqaaiabbMgaPbqabaGccqGHRaWkcqaIXaqmcqGGPaqkaaaabaaabaGaeeizaqMaeeOla4IaeeOzayMaeeOla4IaeeiiaaIaeeypa0JaeeiiaaIae8xVd4Maey4kaSIae4NBa42aaSbaaSqaaiabbMgaPbqabaGccqGHRaWkcqaIXaqmaeaacaWLjaGaaCzcamaabmaabaGaeG4naCdacaGLOaGaayzkaaaaaaaa@89E2@

Intuitively, this distribution represents "shrunk" estimates of the mean and variance. mi2
 MathType@MTEF@5@5@+=feaafiart1ev1aaatCvAUfKttLearuWrP9MDH5MBPbIqV92AaeXatLxBI9gBaebbnrfifHhDYfgasaacH8akY=wiFfYdH8Gipec8Eeeu0xXdbba9frFj0=OqFfea0dXdd9vqai=hGuQ8kuc9pgc9s8qqaq=dirpe0xb9q8qiLsFr0=vr0=vr0dc8meaabaqaciaacaGaaeqabaqabeGadaaakeaacqWGTbqBdaqhaaWcbaGaemyAaKgabaGaeGOmaidaaaaa@3089@ appears in the estimate of the variance σi2
 MathType@MTEF@5@5@+=feaafiart1ev1aaatCvAUfKttLearuWrP9MDH5MBPbIqV92AaeXatLxBI9gBaebbnrfifHhDYfgasaacH8akY=wiFfYdH8Gipec8Eeeu0xXdbba9frFj0=OqFfea0dXdd9vqai=hGuQ8kuc9pgc9s8qqaq=dirpe0xb9q8qiLsFr0=vr0=vr0dc8meaabaqaciaacaGaaeqabaqabeGadaaakeaaiiGacqWFdpWCdaqhaaWcbaGaemyAaKgabaGaeGOmaidaaaaa@30F0@ because mi2
 MathType@MTEF@5@5@+=feaafiart1ev1aaatCvAUfKttLearuWrP9MDH5MBPbIqV92AaeXatLxBI9gBaebbnrfifHhDYfgasaacH8akY=wiFfYdH8Gipec8Eeeu0xXdbba9frFj0=OqFfea0dXdd9vqai=hGuQ8kuc9pgc9s8qqaq=dirpe0xb9q8qiLsFr0=vr0=vr0dc8meaabaqaciaacaGaaeqabaqabeGadaaakeaacqWGTbqBdaqhaaWcbaGaemyAaKgabaGaeGOmaidaaaaa@3089@ contains information about the variance (in our model the expectation of μi2
 MathType@MTEF@5@5@+=feaafiart1ev1aaatCvAUfKttLearuWrP9MDH5MBPbIqV92AaeXatLxBI9gBaebbnrfifHhDYfgasaacH8akY=wiFfYdH8Gipec8Eeeu0xXdbba9frFj0=OqFfea0dXdd9vqai=hGuQ8kuc9pgc9s8qqaq=dirpe0xb9q8qiLsFr0=vr0=vr0dc8meaabaqaciaacaGaaeqabaqabeGadaaakeaaiiGacqWF8oqBdaqhaaWcbaGaemyAaKgabaGaeGOmaidaaaaa@30E3@ is σi2
 MathType@MTEF@5@5@+=feaafiart1ev1aaatCvAUfKttLearuWrP9MDH5MBPbIqV92AaeXatLxBI9gBaebbnrfifHhDYfgasaacH8akY=wiFfYdH8Gipec8Eeeu0xXdbba9frFj0=OqFfea0dXdd9vqai=hGuQ8kuc9pgc9s8qqaq=dirpe0xb9q8qiLsFr0=vr0=vr0dc8meaabaqaciaacaGaaeqabaqabeGadaaakeaaiiGacqWFdpWCdaqhaaWcbaGaemyAaKgabaGaeGOmaidaaaaa@30F0@/*β*). The degrees of freedom for this *t *distribution includes both the observations *n*_*i *_and the prior knowledge about the variance *ν*.

Given this posterior distribution, we can use the standard *t *test to answer questions about the confidence of measurement for gene *i*, e.g., to give a 95% confidence interval for the log-change *μ*_*i *_or the posterior probability that the gene went up (*P*(*μ*_*i *_> 0)).

### Accounting for systematic errors

The key advantage of our approach is to use biological knowledge (i.e., operon predictions) to take systematic errors into account. By definition, these systematic errors will not be eliminated by increasing the number of replicate measurements, but their size can be estimated from the variation between genes in the same operon. In this section, we add systematic errors to the above model (*γ *< ∞, *ε*_*i *_≠ 0) and describe how to account for such bias. Specifically, we show how to estimate the amount of bias and how take the bias into account when assessing significance.

#### Estimating the parameters

If we ignore the distinction between systematic error *ε*_*i *_and true variation *μ*_*i*_, then we can replace *μ*_*i *_with μi'
 MathType@MTEF@5@5@+=feaafiart1ev1aaatCvAUfKttLearuWrP9MDH5MBPbIqV92AaeXatLxBI9gBaebbnrfifHhDYfgasaacH8akY=wiFfYdH8Gipec8Eeeu0xXdbba9frFj0=OqFfea0dXdd9vqai=hGuQ8kuc9pgc9s8qqaq=dirpe0xb9q8qiLsFr0=vr0=vr0dc8meaabaqaciaacaGaaeqabaqabeGadaaakeaaiiGacqWF8oqBdaqhaaWcbaGaemyAaKgabaGaei4jaCcaaaaa@30C7@ ≡ *μ*_*i *_+ *ε*_*i*_. The distribution of μi'
 MathType@MTEF@5@5@+=feaafiart1ev1aaatCvAUfKttLearuWrP9MDH5MBPbIqV92AaeXatLxBI9gBaebbnrfifHhDYfgasaacH8akY=wiFfYdH8Gipec8Eeeu0xXdbba9frFj0=OqFfea0dXdd9vqai=hGuQ8kuc9pgc9s8qqaq=dirpe0xb9q8qiLsFr0=vr0=vr0dc8meaabaqaciaacaGaaeqabaqabeGadaaakeaaiiGacqWF8oqBdaqhaaWcbaGaemyAaKgabaGaei4jaCcaaaaa@30C7@ is given by

μi'~N(0,1θiβ)+N(0,1θiγ)=N(0,1θi⋅(1β+1γ))=N(0,1θiβ′)     (8)
 MathType@MTEF@5@5@+=feaafiart1ev1aaatCvAUfKttLearuWrP9MDH5MBPbIqV92AaeXatLxBI9gBaebbnrfifHhDYfgasaacH8akY=wiFfYdH8Gipec8Eeeu0xXdbba9frFj0=OqFfea0dXdd9vqai=hGuQ8kuc9pgc9s8qqaq=dirpe0xb9q8qiLsFr0=vr0=vr0dc8meaabaqaciaacaGaaeqabaqabeGadaaakeaafaqabeGacaaabaacciGae8hVd02aa0baaSqaaiabdMgaPbqaaiabcEcaNaaakiabc6ha+jabd6eaonaabmaabaGaeGimaaJaeiilaWYaaSaaaeaacqaIXaqmaeaacqWF4oqCdaWgaaWcbaGaemyAaKgabeaakiab=j7aIbaaaiaawIcacaGLPaaacqGHRaWkcqWGobGtdaqadaqaaiabicdaWiabcYcaSmaalaaabaGaeGymaedabaGae8hUde3aaSbaaSqaaiabdMgaPbqabaGccqWFZoWzaaaacaGLOaGaayzkaaaabaaabaGaeyypa0JaemOta40aaeWaaeaacqaIWaamcqGGSaaldaWcaaqaaiabigdaXaqaaiab=H7aXnaaBaaaleaacqWGPbqAaeqaaaaakiabgwSixlabcIcaOmaalaaabaGaeGymaedabaGae8NSdigaaiabgUcaRmaalaaabaGaeGymaedabaGae83SdCgaaiabcMcaPaGaayjkaiaawMcaaiabg2da9iabd6eaonaabmaabaGaeGimaaJaeiilaWYaaSaaaeaacqaIXaqmaeaacqWF4oqCdaWgaaWcbaacbiGae4xAaKgabeaakiqb=j7aIzaafaaaaaGaayjkaiaawMcaaaqaaiaaxMaacaWLjaWaaeWaaeaacqaI4aaoaiaawIcacaGLPaaaaaaaaa@6B1E@

where 1/*β' *≡ 1/*β *+ 1/*γ*, so that the form of the distribution of *m*_*i *_for a model with systematic errors is the same as that for a model without systematic errors, except that we replace *β *with *β'*. The distribution of si2
 MathType@MTEF@5@5@+=feaafiart1ev1aaatCvAUfKttLearuWrP9MDH5MBPbIqV92AaeXatLxBI9gBaebbnrfifHhDYfgasaacH8akY=wiFfYdH8Gipec8Eeeu0xXdbba9frFj0=OqFfea0dXdd9vqai=hGuQ8kuc9pgc9s8qqaq=dirpe0xb9q8qiLsFr0=vr0=vr0dc8meaabaqaciaacaGaaeqabaqabeGadaaakeaacqWGZbWCdaqhaaWcbaGaemyAaKgabaGaeGOmaidaaaaa@3095@ is not affected by systematic errors. Thus, we can estimate *α*, *ν *and *β' *using the method for the simplified model.

We then find the maximum likelihood estimate of *γ*, which controls the amount of bias, by using our assumption that genes in the same operon will have the same values of *μ*_*i *_and of *θ*_*i *_= 1/σi2
 MathType@MTEF@5@5@+=feaafiart1ev1aaatCvAUfKttLearuWrP9MDH5MBPbIqV92AaeXatLxBI9gBaebbnrfifHhDYfgasaacH8akY=wiFfYdH8Gipec8Eeeu0xXdbba9frFj0=OqFfea0dXdd9vqai=hGuQ8kuc9pgc9s8qqaq=dirpe0xb9q8qiLsFr0=vr0=vr0dc8meaabaqaciaacaGaaeqabaqabeGadaaakeaaiiGacqWFdpWCdaqhaaWcbaGaemyAaKgabaGaeGOmaidaaaaa@30F0@. The total likelihood of the data can be decomposed into terms for individual genes and pairwise terms for operon pairs:

f(x→1…x→N)=∏if(x→i)∏ijf(x→i,x→j)f(x→i)⋅f(x→j)     (9)
 MathType@MTEF@5@5@+=feaafiart1ev1aaatCvAUfKttLearuWrP9MDH5MBPbIqV92AaeXatLxBI9gBaebbnrfifHhDYfgasaacH8akY=wiFfYdH8Gipec8Eeeu0xXdbba9frFj0=OqFfea0dXdd9vqai=hGuQ8kuc9pgc9s8qqaq=dirpe0xb9q8qiLsFr0=vr0=vr0dc8meaabaqaciaacaGaaeqabaqabeGadaaakeaacqWGMbGzcqGGOaakcuWG4baEgaWcamaaBaaaleaacqaIXaqmaeqaaOGaeSOjGSKafmiEaGNbaSaadaWgaaWcbaGaemOta4eabeaakiabcMcaPiabg2da9maarafabaGaemOzayMaeiikaGIafmiEaGNbaSaadaWgaaWcbaGaemyAaKgabeaakiabcMcaPmaarafabaWaaSaaaeaacqWGMbGzcqGGOaakcuWG4baEgaWcamaaBaaaleaacqWGPbqAaeqaaOGaeiilaWIafmiEaGNbaSaadaWgaaWcbaGaemOAaOgabeaakiabcMcaPaqaaiabdAgaMjabcIcaOiqbdIha4zaalaWaaSbaaSqaaiabdMgaPbqabaGccqGGPaqkcqGHflY1cqWGMbGzcqGGOaakcuWG4baEgaWcamaaBaaaleaacqWGQbGAaeqaaOGaeiykaKcaaaWcbaGaemyAaKMaemOAaOgabeqdcqGHpis1aaWcbaGaemyAaKgabeqdcqGHpis1aOGaaCzcaiaaxMaadaqadaqaaiabiMda5aGaayjkaiaawMcaaaaa@6266@

We have already taken into account the effect of *γ *on the single-gene likelihoods *f*(x→i
 MathType@MTEF@5@5@+=feaafiart1ev1aaatCvAUfKttLearuWrP9MDH5MBPbIqV92AaeXatLxBI9gBaebbnrfifHhDYfgasaacH8akY=wiFfYdH8Gipec8Eeeu0xXdbba9frFj0=OqFfea0dXdd9vqai=hGuQ8kuc9pgc9s8qqaq=dirpe0xb9q8qiLsFr0=vr0=vr0dc8meaabaqaciaacaGaaeqabaqabeGadaaakeaacuWG4baEgaWcamaaBaaaleaacqWGPbqAaeqaaaaa@2FBE@) by introducing *β'*, which is now being held constant, so these terms do not need to be considered. To derive an equation for the pairwise likelihood ratios, we first note the possibility that the operon prediction is incorrect, in which case the genes are independent and the likelihood ratio is 1:

f(x→i,x→j)f(x→i)⋅f(x→j)=1−P(Operonij)+P(Operonij)⋅f(x→i,x→j|Operonij)f(x→i)⋅f(x→j)     (10)
 MathType@MTEF@5@5@+=feaafiart1ev1aaatCvAUfKttLearuWrP9MDH5MBPbIqV92AaeXatLxBI9gBaebbnrfifHhDYfgasaacH8akY=wiFfYdH8Gipec8Eeeu0xXdbba9frFj0=OqFfea0dXdd9vqai=hGuQ8kuc9pgc9s8qqaq=dirpe0xb9q8qiLsFr0=vr0=vr0dc8meaabaqaciaacaGaaeqabaqabeGadaaakqGabeqaaG0abaWaaSaaaeaacqWGMbGzcqGGOaakcuWG4baEgaWcamaaBaaaleaacqWGPbqAaeqaaOGaeiilaWIafmiEaGNbaSaadaWgaaWcbaGaemOAaOgabeaakiabcMcaPaqaaiabdAgaMjabcIcaOiqbdIha4zaalaWaaSbaaSqaaiabdMgaPbqabaGccqGGPaqkcqGHflY1cqWGMbGzcqGGOaakcuWG4baEgaWcamaaBaaaleaacqWGQbGAaeqaaOGaeiykaKcaaiabg2da9iabigdaXiabgkHiTiabdcfaqjabcIcaOiabd+eapjabdchaWjabdwgaLjabdkhaYjabd+gaVjabd6gaUnaaBaaaleaacqWGPbqAcqWGQbGAaeqaaOGaeiykaKcabaGaaCzcaiabgUcaRiabdcfaqjabcIcaOiabd+eapjabdchaWjabdwgaLjabdkhaYjabd+gaVjabd6gaUnaaBaaaleaacqWGPbqAcqWGQbGAaeqaaOGaeiykaKIaeyyXIC9aaSaaaeaacqWGMbGzcqGGOaakcuWG4baEgaWcamaaBaaaleaacqWGPbqAaeqaaOGaeiilaWIafmiEaGNbaSaadaWgaaWcbaGaemOAaOgabeaakmaaeeaabaGaem4ta8KaemiCaaNaemyzauMaemOCaiNaem4Ba8MaemOBa42aaSbaaSqaaiabdMgaPjabdQgaQbqabaGccqGGPaqkaiaawEa7aaqaaiabdAgaMjabcIcaOiqbdIha4zaalaWaaSbaaSqaaiabdMgaPbqabaGccqGGPaqkcqGHflY1cqWGMbGzcqGGOaakcuWG4baEgaWcamaaBaaaleaacqWGQbGAaeqaaOGaeiykaKcaaiaaxMaacaWLjaWaaeWaaeaacqaIXaqmcqaIWaamaiaawIcacaGLPaaaaaaa@924B@

The pairwise likelihood ratio for the operon case can be derived from

f(x→i,x→j|Operonij)=∫0∞dθijf(θij)∫−∞∞dμijf(μij)⋅f(mi,si2|μij,θij)⋅f(mj,sj2|μij,θij)     (11)
 MathType@MTEF@5@5@+=feaafiart1ev1aaatCvAUfKttLearuWrP9MDH5MBPbIqV92AaeXatLxBI9gBaebbnrfifHhDYfgasaacH8akY=wiFfYdH8Gipec8Eeeu0xXdbba9frFj0=OqFfea0dXdd9vqai=hGuQ8kuc9pgc9s8qqaq=dirpe0xb9q8qiLsFr0=vr0=vr0dc8meaabaqaciaacaGaaeqabaqabeGadaaakeaafaqabeGacaaabaGaemOzay2aaeWaaeaacuWG4baEgaWcamaaBaaaleaacqWGPbqAaeqaaOGaeiilaWIafmiEaGNbaSaadaWgaaWcbaGaemOAaOgabeaakmaaeeaabaGaem4ta8KaemiCaaNaemyzauMaemOCaiNaem4Ba8MaemOBa42aaSbaaSqaaiabdMgaPjabdQgaQbqabaaakiaawEa7aaGaayjkaiaawMcaaiabg2da9maapedabaGaemizaqgcciGae8hUde3aaSbaaSqaaiabdMgaPjabdQgaQbqabaGccqWGMbGzdaqadaqaaiab=H7aXnaaBaaaleaacqWGPbqAcqWGQbGAaeqaaaGccaGLOaGaayzkaaWaa8qmaeaacqWGKbazcqWF8oqBdaWgaaWcbaGaemyAaKMaemOAaOgabeaakiabdAgaMnaabmaabaGae8hVd02aaSbaaSqaaiabdMgaPjabdQgaQbqabaaakiaawIcacaGLPaaaaSqaaiabgkHiTiab=5HiLcqaaiab=5HiLcqdcqGHRiI8aaWcbaGaeGimaadabaGae8NhIukaniabgUIiYdaakeaaaeaacqGHflY1cqWGMbGzdaqadaqaaiabd2gaTnaaBaaaleaacqWGPbqAaeqaaOGaeiilaWIaem4Cam3aa0baaSqaaiabdMgaPbqaaiabikdaYaaakmaaeeaabaGae8hVd02aaSbaaSqaaiabdMgaPjabdQgaQbqabaGccqGGSaalcqWF4oqCdaWgaaWcbaGaemyAaKMaemOAaOgabeaaaOGaay5bSdaacaGLOaGaayzkaaGaeyyXICTaemOzay2aaeWaaeaacqWGTbqBdaWgaaWcbaGaemOAaOgabeaakiabcYcaSiabdohaZnaaDaaaleaacqWGQbGAaeaacqaIYaGmaaGcdaabbaqaaiab=X7aTnaaBaaaleaacqWGPbqAcqWGQbGAaeqaaOGaeiilaWIae8hUde3aaSbaaSqaaiabdMgaPjabdQgaQbqabaaakiaawEa7aaGaayjkaiaawMcaaaqaaiaaxMaacaWLjaWaaeWaaeaacqaIXaqmcqaIXaqmaiaawIcacaGLPaaaaaaaaa@9FA6@

f(x→i)=∫0∞dθif(θi)∫−∞∞dμif(μi)⋅f(mi,si2|μi,θi)     (12)
 MathType@MTEF@5@5@+=feaafiart1ev1aaatCvAUfKttLearuWrP9MDH5MBPbIqV92AaeXatLxBI9gBaebbnrfifHhDYfgasaacH8akY=wiFfYdH8Gipec8Eeeu0xXdbba9frFj0=OqFfea0dXdd9vqai=hGuQ8kuc9pgc9s8qqaq=dirpe0xb9q8qiLsFr0=vr0=vr0dc8meaabaqaciaacaGaaeqabaqabeGadaaakeaacqWGMbGzdaqadaqaaiqbdIha4zaalaWaaSbaaSqaaiabdMgaPbqabaaakiaawIcacaGLPaaacqGH9aqpdaWdXaqaaiabdsgaKHGaciab=H7aXnaaBaaaleaacqWGPbqAaeqaaOGaemOzay2aaeWaaeaacqWF4oqCdaWgaaWcbaGaemyAaKgabeaaaOGaayjkaiaawMcaaaWcbaGaeGimaadabaGae8NhIukaniabgUIiYdGcdaWdXaqaaiabdsgaKjab=X7aTnaaBaaaleaacqWGPbqAaeqaaOGaemOzay2aaeWaaeaacqWF8oqBdaWgaaWcbaGaemyAaKgabeaaaOGaayjkaiaawMcaaiabgwSixlabdAgaMnaabmaabaGaemyBa02aaSbaaSqaaiabdMgaPbqabaGccqGGSaalcqWGZbWCdaqhaaWcbaGaemyAaKgabaGaeGOmaidaaOWaaqqaaeaacqWF8oqBdaWgaaWcbaGaemyAaKgabeaakiabcYcaSiab=H7aXnaaBaaaleaacqWGPbqAaeqaaaGccaGLhWoaaiaawIcacaGLPaaaaSqaaiab=jHiTiab=5HiLcqaaiab=5HiLcqdcqGHRiI8aOGaaCzcaiaaxMaadaqadaqaaiabigdaXiabikdaYaGaayjkaiaawMcaaaaa@6E2C@

f(mi,si2|μi,θi)=∫−∞∞dεif(εi)⋅f(mi,si2|μi,θi,εi)     (13)
 MathType@MTEF@5@5@+=feaafiart1ev1aaatCvAUfKttLearuWrP9MDH5MBPbIqV92AaeXatLxBI9gBaebbnrfifHhDYfgasaacH8akY=wiFfYdH8Gipec8Eeeu0xXdbba9frFj0=OqFfea0dXdd9vqai=hGuQ8kuc9pgc9s8qqaq=dirpe0xb9q8qiLsFr0=vr0=vr0dc8meaabaqaciaacaGaaeqabaqabeGadaaakeaacqWGMbGzdaqadaqaaiabd2gaTnaaBaaaleaacqWGPbqAaeqaaOGaeiilaWIaem4Cam3aa0baaSqaaiabdMgaPbqaaiabikdaYaaakmaaeeaabaacciGae8hVd02aaSbaaSqaaiabdMgaPbqabaGccqGGSaalcqWF4oqCdaWgaaWcbaGaemyAaKgabeaaaOGaay5bSdaacaGLOaGaayzkaaGaeyypa0Zaa8qmaeaacqWGKbazcqWF1oqzdaWgaaWcbaacbiGae4xAaKgabeaakiabdAgaMnaabmaabaGae8xTdu2aaSbaaSqaaiabdMgaPbqabaaakiaawIcacaGLPaaaaSqaaiab=jHiTiab=5HiLcqaaiab=5HiLcqdcqGHRiI8aOGaeyyXICTaemOzay2aaeWaaeaacqWGTbqBdaWgaaWcbaGaemyAaKgabeaakiabcYcaSiabdohaZnaaDaaaleaacqWGPbqAaeaacqaIYaGmaaGcdaabbaqaaiab=X7aTnaaBaaaleaacqWGPbqAaeqaaOGaeiilaWIae8hUde3aaSbaaSqaaiabdMgaPbqabaGccqGGSaalcqWF1oqzdaWgaaWcbaGaemyAaKgabeaaaOGaay5bSdaacaGLOaGaayzkaaGaaCzcaiaaxMaadaqadaqaaiabigdaXiabiodaZaGaayjkaiaawMcaaaaa@708E@

to give

f(x→i,x→j|Operonij)f(x→i)⋅f(x→j)=(α2)−ν+12⋅γ(Ni+γ)⋅(Nj+γ)⋅ββ+Ni'+Nj'⋅(β'+Ni)(β'+Nj)β'⋅Γ(ν+ni+nj+12)Γ(ν+12)Γ(ν+ni+12)Γ(ν+nj+12)⋅(Xij/2)−ν+ni+nj+12(Xi/2)−−ν+ni+12⋅(Xj/2)−ν+nj+12     (14)
 MathType@MTEF@5@5@+=feaafiart1ev1aaatCvAUfKttLearuWrP9MDH5MBPbIqV92AaeXatLxBI9gBaebbnrfifHhDYfgasaacH8akY=wiFfYdH8Gipec8Eeeu0xXdbba9frFj0=OqFfea0dXdd9vqai=hGuQ8kuc9pgc9s8qqaq=dirpe0xb9q8qiLsFr0=vr0=vr0dc8meaabaqaciaacaGaaeqabaqabeGadaaakqGabeqaaG3abaWaaSaaaeaacqWGMbGzdaqadaqaaiqbdIha4zaalaWaaSbaaSqaaiabdMgaPbqabaGccqGGSaalcuWG4baEgaWcamaaBaaaleaacqWGQbGAaeqaaOWaaqqaaeaacqWGpbWtcqWGWbaCcqWGLbqzcqWGYbGCcqWGVbWBcqWGUbGBdaWgaaWcbaGaemyAaKMaemOAaOgabeaaaOGaay5bSdaacaGLOaGaayzkaaaabaGaemOzay2aaeWaaeaacuWG4baEgaWcamaaBaaaleaacqWGPbqAaeqaaaGccaGLOaGaayzkaaGaeyyXICTaemOzay2aaeWaaeaacuWG4baEgaWcamaaBaaaleaacqWGQbGAaeqaaaGccaGLOaGaayzkaaaaaiabg2da9maabmaabaWaaSaaaeaaiiGacqWFXoqyaeaacqaIYaGmaaaacaGLOaGaayzkaaWaaWbaaSqabeaacqGHsisldaWcbaadbaGae8xVd4Maey4kaSIaeGymaedabaGaeGOmaidaaaaakiabgwSixpaalaaabaGae83SdCgabaWaaOaaaeaacqGGOaakcqWGobGtdaWgaaWcbaGaemyAaKgabeaakiabgUcaRiab=n7aNjabcMcaPiabgwSixlabcIcaOiabd6eaonaaBaaaleaacqWGQbGAaeqaaOGaey4kaSIae83SdCMaeiykaKcaleqaaaaaaOqaaiaaxMaacqGHflY1daGcaaqaamaalaaabaGae8NSdigabaGae8NSdiMaey4kaSIaemOta40aa0baaSqaaiabdMgaPbqaaiabcEcaNaaakiabgUcaRiabd6eaonaaDaaaleaacqWGQbGAaeaacqGGNaWjaaaaaaqabaGccqGHflY1daWcaaqaamaakaaabaGaeiikaGIae8NSdiMaei4jaCIaey4kaSIaemOta40aaSbaaSqaaiabdMgaPbqabaGccqGGPaqkcqGGOaakcqWFYoGycqGGNaWjcqGHRaWkcqWGobGtdaWgaaWcbaGaemOAaOgabeaakiabcMcaPaWcbeaaaOqaaiab=j7aIjabcEcaNaaaaeaacqGHflY1daWcaaqaaiabfo5ahnaabmaabaWaaSqaaSqaaiab=17aUjabgUcaRiabd6gaUnaaBaaameaacqWGPbqAaeqaaSGaey4kaSIaemOBa42aaSbaaWqaaiabdQgaQbqabaWccqGHRaWkcqaIXaqmaeaacqaIYaGmaaaakiaawIcacaGLPaaacqqHtoWrdaqadaqaamaaleaaleaacqWF9oGBcqGHRaWkcqaIXaqmaeaacqaIYaGmaaaakiaawIcacaGLPaaaaeaacqqHtoWrdaqadaqaamaaleaaleaacqWF9oGBcqGHRaWkcqWGUbGBdaWgaaadbaGaemyAaKgabeaaliabgUcaRiabigdaXaqaaiabikdaYaaaaOGaayjkaiaawMcaaiabfo5ahnaabmaabaWaaSqaaSqaaiab=17aUjabgUcaRiabd6gaUnaaBaaameaacqWGQbGAaeqaaSGaey4kaSIaeGymaedabaGaeGOmaidaaaGccaGLOaGaayzkaaaaaiabgwSixpaalaaabaWaaeWaaeaacqWGybawdaWgaaWcbaGaemyAaKMaemOAaOgabeaakiabc+caViabikdaYaGaayjkaiaawMcaamaaCaaaleqabaGaeyOeI0YaaSqaaWqaaiab=17aUjabgUcaRiabd6gaUnaaBaaabaGaemyAaKgabeaacqGHRaWkcqWGUbGBdaWgaaqaaiabdQgaQbqabaGaey4kaSIaeGymaedabaGaeGOmaidaaaaaaOqaamaabmaabaGaemiwaG1aaSbaaSqaaiabdMgaPbqabaGccqGGVaWlcqaIYaGmaiaawIcacaGLPaaadaahaaWcbeqaaiabgkHiTmaaleaameaacqGHsislcqWF9oGBcqGHRaWkcqWGUbGBdaWgaaqaaiabdMgaPbqabaGaey4kaSIaeGymaedabaGaeGOmaidaaaaakiabgwSixpaabmaabaGaemiwaG1aaSbaaSqaaiabdQgaQbqabaGccqGGVaWlcqaIYaGmaiaawIcacaGLPaaadaahaaWcbeqaaiabgkHiTmaaleaameaacqWF9oGBcqGHRaWkcqWGUbGBdaWgaaqaaiabdQgaQbqabaGaey4kaSIaeGymaedabaGaeGOmaidaaaaaaaGccaWLjaGaaCzcamaabmaabaGaeGymaeJaeGinaqdacaGLOaGaayzkaaaaaaa@0335@

where

Xi=α+si2+mj2⋅Ni⋅β′β′+Ni     (15)
 MathType@MTEF@5@5@+=feaafiart1ev1aaatCvAUfKttLearuWrP9MDH5MBPbIqV92AaeXatLxBI9gBaebbnrfifHhDYfgasaacH8akY=wiFfYdH8Gipec8Eeeu0xXdbba9frFj0=OqFfea0dXdd9vqai=hGuQ8kuc9pgc9s8qqaq=dirpe0xb9q8qiLsFr0=vr0=vr0dc8meaabaqaciaacaGaaeqabaqabeGadaaakeaacqWGybawdaWgaaWcbaGaemyAaKgabeaakiabg2da9GGaciab=f7aHjab=TcaRiabdohaZnaaDaaaleaacqWGPbqAaeaacqaIYaGmaaGccqGHRaWkcqWGTbqBdaqhaaWcbaGaemOAaOgabaGaeGOmaidaaOGaeyyXICTaemOta40aaSbaaSqaaiabdMgaPbqabaGccqGHflY1daWcaaqaaiqb=j7aIzaafaaabaGaf8NSdiMbauaacqGHRaWkcqWGobGtdaWgaaWcbaGaemyAaKgabeaaaaGccaWLjaGaaCzcamaabmaabaGaeGymaeJaeGynaudacaGLOaGaayzkaaaaaa@4EB2@

and similarly for j, and

Xij=α+si2+sj2+Ni'⋅mi2+Nj'⋅mj2−(mi⋅Ni'+mj⋅Nj')2β+Ni'+Nj'     (16)
 MathType@MTEF@5@5@+=feaafiart1ev1aaatCvAUfKttLearuWrP9MDH5MBPbIqV92AaeXatLxBI9gBaebbnrfifHhDYfgasaacH8akY=wiFfYdH8Gipec8Eeeu0xXdbba9frFj0=OqFfea0dXdd9vqai=hGuQ8kuc9pgc9s8qqaq=dirpe0xb9q8qiLsFr0=vr0=vr0dc8meaabaqaciaacaGaaeqabaqabeGadaaakeaacqWGybawdaWgaaWcbaGaemyAaKMaemOAaOgabeaakiabg2da9GGaciab=f7aHjabgUcaRiabdohaZnaaDaaaleaacqWGPbqAaeaacqaIYaGmaaGccqGHRaWkcqWGZbWCdaqhaaWcbaGaemOAaOgabaGaeGOmaidaaOGaey4kaSIaemOta40aa0baaSqaaiabdMgaPbqaaiabcEcaNaaakiabgwSixlabd2gaTnaaDaaaleaacqWGPbqAaeaacqaIYaGmaaGccqGHRaWkcqWGobGtdaqhaaWcbaGaemOAaOgabaGaei4jaCcaaOGaeyyXICTaemyBa02aa0baaSqaaiabdQgaQbqaaiabikdaYaaakiabgkHiTmaalaaabaWaaeWaaeaacqWGTbqBdaWgaaWcbaGaemyAaKgabeaakiabgwSixlabd6eaonaaDaaaleaacqWGPbqAaeaacqGGNaWjaaGccqGHRaWkcqWGTbqBdaWgaaWcbaGaemOAaOgabeaakiabgwSixlabd6eaonaaDaaaleaacqWGQbGAaeaacqGGNaWjaaaakiaawIcacaGLPaaadaahaaWcbeqaaiabikdaYaaaaOqaaiab=j7aIjabgUcaRiabd6eaonaaDaaaleaacqWGPbqAaeaacqGGNaWjaaGccqGHRaWkcqWGobGtdaqhaaWcbaGaemOAaOgabaGaei4jaCcaaaaakiaaxMaacaWLjaWaaeWaaeaacqaIXaqmcqaI2aGnaiaawIcacaGLPaaaaaa@77CD@

and

Ni'≡(Ni−1+γ−1)−1     (17)
 MathType@MTEF@5@5@+=feaafiart1ev1aaatCvAUfKttLearuWrP9MDH5MBPbIqV92AaeXatLxBI9gBaebbnrfifHhDYfgasaacH8akY=wiFfYdH8Gipec8Eeeu0xXdbba9frFj0=OqFfea0dXdd9vqai=hGuQ8kuc9pgc9s8qqaq=dirpe0xb9q8qiLsFr0=vr0=vr0dc8meaabaqaciaacaGaaeqabaqabeGadaaakeaacqWGobGtdaqhaaWcbaGaemyAaKgabaGaei4jaCcaaOGaeyyyIO7aaeWaaeaacqWGobGtdaqhaaWcbaGaemyAaKgabaGaeyOeI0IaeGymaedaaOGaey4kaSccciGae83SdC2aaWbaaSqabeaacqGHsislcqaIXaqmaaaakiaawIcacaGLPaaadaahaaWcbeqaaiabgkHiTiabigdaXaaakiaaxMaacaWLjaWaaeWaaeaacqaIXaqmcqaI3aWnaiaawIcacaGLPaaaaaa@4390@

and similarly for j. Although much of Eq. 14 has no simple intuitive explanation, and unfortunately the constant terms are required (e.g. see Eq. 10), the (*X*/2)^-*df*/2 ^terms can be viewed as *t *distribution forms for the joint probability *f*(x→i
 MathType@MTEF@5@5@+=feaafiart1ev1aaatCvAUfKttLearuWrP9MDH5MBPbIqV92AaeXatLxBI9gBaebbnrfifHhDYfgasaacH8akY=wiFfYdH8Gipec8Eeeu0xXdbba9frFj0=OqFfea0dXdd9vqai=hGuQ8kuc9pgc9s8qqaq=dirpe0xb9q8qiLsFr0=vr0=vr0dc8meaabaqaciaacaGaaeqabaqabeGadaaakeaacuWG4baEgaWcamaaBaaaleaacqWGPbqAaeqaaaaa@2FBE@, x→j
 MathType@MTEF@5@5@+=feaafiart1ev1aaatCvAUfKttLearuWrP9MDH5MBPbIqV92AaeXatLxBI9gBaebbnrfifHhDYfgasaacH8akY=wiFfYdH8Gipec8Eeeu0xXdbba9frFj0=OqFfea0dXdd9vqai=hGuQ8kuc9pgc9s8qqaq=dirpe0xb9q8qiLsFr0=vr0=vr0dc8meaabaqaciaacaGaaeqabaqabeGadaaakeaacuWG4baEgaWcamaaBaaaleaacqWGQbGAaeqaaaaa@2FC0@|*Operon*_*ij*_) divided by similar forms for the independent probabilities *f*(x→i
 MathType@MTEF@5@5@+=feaafiart1ev1aaatCvAUfKttLearuWrP9MDH5MBPbIqV92AaeXatLxBI9gBaebbnrfifHhDYfgasaacH8akY=wiFfYdH8Gipec8Eeeu0xXdbba9frFj0=OqFfea0dXdd9vqai=hGuQ8kuc9pgc9s8qqaq=dirpe0xb9q8qiLsFr0=vr0=vr0dc8meaabaqaciaacaGaaeqabaqabeGadaaakeaacuWG4baEgaWcamaaBaaaleaacqWGPbqAaeqaaaaa@2FBE@) and *f*(x→j
 MathType@MTEF@5@5@+=feaafiart1ev1aaatCvAUfKttLearuWrP9MDH5MBPbIqV92AaeXatLxBI9gBaebbnrfifHhDYfgasaacH8akY=wiFfYdH8Gipec8Eeeu0xXdbba9frFj0=OqFfea0dXdd9vqai=hGuQ8kuc9pgc9s8qqaq=dirpe0xb9q8qiLsFr0=vr0=vr0dc8meaabaqaciaacaGaaeqabaqabeGadaaakeaacuWG4baEgaWcamaaBaaaleaacqWGQbGAaeqaaaaa@2FC0@).

Given this solution for the likelihood of the data, we can use a Newton iteration method to find the value of *γ *that maximizes the product of the pair-wise likelihood ratios given by Eq. 10.

#### Significance of individual genes

If we ignore the information from other genes, then the posterior distribution of *μ*_*i *_is given by a *t *distribution with

mean =mi⋅Ni'β+Ni'variance = α+si2+mi2⋅Ni'⋅β/(Ni'+β)(β+Ni')⋅(ν+ni+1)d.f. = ν+ni+1     (18)
 MathType@MTEF@5@5@+=feaafiart1ev1aaatCvAUfKttLearuWrP9MDH5MBPbIqV92AaeXatLxBI9gBaebbnrfifHhDYfgasaacH8akY=wiFfYdH8Gipec8Eeeu0xXdbba9frFj0=OqFfea0dXdd9vqai=hGuQ8kuc9pgc9s8qqaq=dirpe0xb9q8qiLsFr0=vr0=vr0dc8meaabaqaciaacaGaaeqabaqabeGadaaakeaafaqabeWacaaabaGaeeyBa0MaeeyzauMaeeyyaeMaeeOBa4MaeeiiaaIaeeypa0ZaaSaaaeaacqqGTbqBdaWgaaWcbaGaeeyAaKgabeaakiabgwSixlabb6eaonaaDaaaleaacqqGPbqAaeaacqqGNaWjaaaakeaaiiGacqWFYoGycqGHRaWkieaacqGFobGtdaqhaaWcbaGae4xAaKgabaWexLMBbXgBcf2CPn2qVrwzqf2zLnharyGvLjhzH5wyaGabaiaa9DcaaaaaaaGcbaaabaGaeeODayNaeeyyaeMaeeOCaiNaeeyAaKMaeeyyaeMaeeOBa4Maee4yamMaeeyzauMaeeiiaaIaeeypa0JaeeiiaaYaaSaaaeaacqWFXoqycqGHRaWkcqGFZbWCdaqhaaWcbaGae4xAaKgabaGae4NmaidaaOGaey4kaSIaeeyBa02aa0baaSqaaiabbMgaPbqaaiabbkdaYaaakiabgwSixlabb6eaonaaDaaaleaacqqGPbqAaeaacqqGNaWjaaGccqGHflY1cqWFYoGycqqGVaWldaqadaqaaiabb6eaonaaDaaaleaacqqGPbqAaeaacqqGNaWjaaGccqGHRaWkcqWFYoGyaiaawIcacaGLPaaaaeaadaqadaqaaiab=j7aIjabgUcaRiab+5eaonaaDaaaleaacqGFPbqAaeaacaqFNaaaaaGccaGLOaGaayzkaaGaeyyXIC9aaeWaaeaacqWF9oGBcqGHRaWkcqGFUbGBdaWgaaWcbaGae4xAaKgabeaakiabgUcaRiabigdaXaGaayjkaiaawMcaaaaaaeaaaeaacqqGKbazcqqGUaGlcqqGMbGzcqqGUaGlcqqGGaaicqqG9aqpcqqGGaaicqWF9oGBcqGHRaWkcqGFUbGBdaWgaaWcbaGaeeyAaKgabeaakiabgUcaRiabigdaXaqaaiaaxMaacaWLjaWaaeWaaeaacqaIXaqmcqaI4aaoaiaawIcacaGLPaaaaaaaaa@9D5E@

This is the same as case without systematic bias except that *N*_*i*_, which describes the amount of data and hence the reduction in uncertainty due to replication, has been replaced by the smaller term Ni'
 MathType@MTEF@5@5@+=feaafiart1ev1aaatCvAUfKttLearuWrP9MDH5MBPbIqV92AaeXatLxBI9gBaebbnrfifHhDYfgasaacH8akY=wiFfYdH8Gipec8Eeeu0xXdbba9frFj0=OqFfea0dXdd9vqai=hGuQ8kuc9pgc9s8qqaq=dirpe0xb9q8qiLsFr0=vr0=vr0dc8meaabaqaciaacaGaaeqabaqabeGadaaakeaacqWGobGtdaqhaaWcbaGaemyAaKgabaGaei4jaCcaaaaa@302F@.

### Significance taking operons into account

Although the method as described so far uses operon predictions to estimate the hyperparameters, it uses only the information for each gene when computing *p*-values. We will refer to these as "single-gene" *p*-values. In this section, we describe 'operon-wise" *p*-values that use information from other genes in the same operon to improve our estimates of the significance of each gene. As we will show in the Results, using this additional information often allows increased confidence in the measurements.

First, assume that we have two genes *i *and *j *that are known to be in the same operon, with the same (unknown) *μ*_*ij *_and *θ*_*ij *_but with differing biases *ε*_*i*_, *ε*_*j*_. Given measurements for the two genes, the posterior distribution for *μ*_*ij *_is a *t *distribution with

mean =Ni'⋅mi+Nj'⋅mjβ+Ni'+Nj'variance = α+si2+sj2+Ni'⋅mi2+Nj'⋅mj2−(Ni⋅mi+Nj⋅mj)2β+Ni'+Nj'(ν+ni+nj+1)⋅(β+Ni'+Nj')d.f. =ν+ni+nj+1     (19)
 MathType@MTEF@5@5@+=feaafiart1ev1aaatCvAUfKttLearuWrP9MDH5MBPbIqV92AaeXatLxBI9gBaebbnrfifHhDYfgasaacH8akY=wiFfYdH8Gipec8Eeeu0xXdbba9frFj0=OqFfea0dXdd9vqai=hGuQ8kuc9pgc9s8qqaq=dirpe0xb9q8qiLsFr0=vr0=vr0dc8meaabaqaciaacaGaaeqabaqabeGadaaakeaafaqabeWacaaabaGaeeyBa0MaeeyzauMaeeyyaeMaeeOBa4MaeeiiaaIaeeypa0ZaaSaaaeaacqqGobGtdaqhaaWcbaGaeeyAaKgabaGaei4jaCcaaOGaeyyXICTaeeyBa02aaSbaaSqaaiabbMgaPbqabaGccqGHRaWkcqqGobGtdaqhaaWcbaGaeeOAaOgabaGaee4jaCcaaOGaeyyXICTaeeyBa02aaSbaaSqaaiabbQgaQbqabaaakeaaiiGacqWFYoGycqGHRaWkcqqGobGtdaqhaaWcbaGaeeyAaKgabaGaee4jaCcaaOGaey4kaSIaeeOta40aa0baaSqaaiabbQgaQbqaaiabbEcaNaaaaaaakeaaaeaacqqG2bGDcqqGHbqycqqGYbGCcqqGPbqAcqqGHbqycqqGUbGBcqqGJbWycqqGLbqzcqqGGaaicqqG9aqpcqqGGaaidaWcaaqaaiab=f7aHjabgUcaRiabbohaZnaaDaaaleaacqqGPbqAaeaacqqGYaGmaaGccqGHRaWkcqqGZbWCdaqhaaWcbaGaeeOAaOgabaGaeeOmaidaaOGaey4kaSIaeeOta40aa0baaSqaaiabbMgaPbqaaiabbEcaNaaakiabgwSixlabb2gaTnaaDaaaleaacqqGPbqAaeaacqqGYaGmaaGccqGHRaWkcqqGobGtdaqhaaWcbaGaeeOAaOgabaGaee4jaCcaaOGaeyyXICTaeeyBa02aa0baaSqaaiabbQgaQbqaaiabbkdaYaaakiabgkHiTmaaleaaleaadaqadaqaaiabb6eaonaaBaaameaacqqGPbqAaeqaaSGaeyyXICTaeeyBa02aaSbaaWqaaiabbMgaPbqabaWccqGHRaWkcqqGobGtdaWgaaadbaGaeeOAaOgabeaaliabgwSixlabb2gaTnaaBaaameaacqqGQbGAaeqaaaWccaGLOaGaayzkaaWaaWbaaWqabeaacqaIYaGmaaaaleaacqWFYoGycqGHRaWkcqqGobGtdaqhaaadbaGaeeyAaKgabaGaee4jaCcaaSGaey4kaSIaeeOta40aa0baaWqaaiabbQgaQbqaaiabbEcaNaaaaaaakeaadaqadaqaaiab=17aUjabgUcaRiabb6gaUnaaBaaaleaacqqGPbqAaeqaaOGaey4kaSIaeeOBa42aaSbaaSqaaiabbQgaQbqabaGccqGHRaWkcqaIXaqmaiaawIcacaGLPaaacqGHflY1daqadaqaaiab=j7aIjabgUcaRiabb6eaonaaDaaaleaacqqGPbqAaeaaieaacqGFNaWjaaGccqGHRaWkcqGFobGtdaqhaaWcbaGae4NAaOgabaGae43jaCcaaaGccaGLOaGaayzkaaaaaaqaaaqaaiabbsgaKjabb6caUiabbAgaMjabb6caUiabbccaGiabb2da9iab=17aUjabgUcaRiab+5gaUnaaBaaaleaacqGFPbqAaeqaaOGaey4kaSIae4NBa42aaSbaaSqaaiab+PgaQbqabaGccqGHRaWkcqaIXaqmaeaacaWLjaGaaCzcamaabmaabaGaeGymaeJaeGyoaKdacaGLOaGaayzkaaaaaaaa@CE9D@

It is straightforward to extend this formula to three or more genes.

In practice, operon predictions are uncertain, and we need to take this uncertainty into account in estimating confidence. We use only the adjacent pairs that are predicted to be in the same operon (those with *P*(*Operon*_*ij*_) ≥ 0.5), as non-adjacent pairs are less reliable. In the most complicated situation, we have genes *i *and *k *on either side of our target gene *j *and four possible cases: singleton transcript *j*, two-gene operon *ij*, two-gene operon *jk*, or three-gene operon *ijk*. The posterior distribution of *μ*_*j *_is then a mixture of the corresponding four posterior distributions, and a specific probability such as *P*(*μ*_*j *_> 0) is determined from a linear combination of the probabilities from four *t *tests.

To determine the weight of the terms in the mixture, we do not use the input probabilities *P*(*Operon*_*ij*_) and *P*(*Operon*_*jk*_). Instead, we use the posterior operon probabilities given the data. That is, we use the microarray data to help estimate the likelihood that a pair of genes are co-transcribed. Using the posterior operon probabilities gives the rigorously correct posterior distribution for *μ*_*j *_(derivation not shown). Using the posterior operon probabilities also prevents the method from asserting that a gene went down when it in fact went up but other genes in the operon went down, because in this situation the posterior probability of the operon will be low.

Using Bayes' law, these posterior probabilities *P*(*Operon*_*ij*_|x→i
 MathType@MTEF@5@5@+=feaafiart1ev1aaatCvAUfKttLearuWrP9MDH5MBPbIqV92AaeXatLxBI9gBaebbnrfifHhDYfgasaacH8akY=wiFfYdH8Gipec8Eeeu0xXdbba9frFj0=OqFfea0dXdd9vqai=hGuQ8kuc9pgc9s8qqaq=dirpe0xb9q8qiLsFr0=vr0=vr0dc8meaabaqaciaacaGaaeqabaqabeGadaaakeaacuWG4baEgaWcamaaBaaaleaacqWGPbqAaeqaaaaa@2FBE@, x→j
 MathType@MTEF@5@5@+=feaafiart1ev1aaatCvAUfKttLearuWrP9MDH5MBPbIqV92AaeXatLxBI9gBaebbnrfifHhDYfgasaacH8akY=wiFfYdH8Gipec8Eeeu0xXdbba9frFj0=OqFfea0dXdd9vqai=hGuQ8kuc9pgc9s8qqaq=dirpe0xb9q8qiLsFr0=vr0=vr0dc8meaabaqaciaacaGaaeqabaqabeGadaaakeaacuWG4baEgaWcamaaBaaaleaacqWGQbGAaeqaaaaa@2FC0@) can be obtained from

P(Operonij|x→i,x→j)P(¬Operonij|x→i,x→j)=P(Operonij)P(¬Operonij)⋅f(x→i,x→j|Operonij)f(x→i)⋅f(x→j)     (20)
 MathType@MTEF@5@5@+=feaafiart1ev1aaatCvAUfKttLearuWrP9MDH5MBPbIqV92AaeXatLxBI9gBaebbnrfifHhDYfgasaacH8akY=wiFfYdH8Gipec8Eeeu0xXdbba9frFj0=OqFfea0dXdd9vqai=hGuQ8kuc9pgc9s8qqaq=dirpe0xb9q8qiLsFr0=vr0=vr0dc8meaabaqaciaacaGaaeqabaqabeGadaaakeaadaWcaaqaaiabdcfaqnaabmaabaGaem4ta8KaemiCaaNaemyzauMaemOCaiNaem4Ba8MaemOBa42aaSbaaSqaaiabdMgaPjabdQgaQbqabaGcdaabbaqaaiqbdIha4zaalaWaaSbaaSqaaiabdMgaPbqabaGccqGGSaalcuWG4baEgaWcamaaBaaaleaacqWGQbGAaeqaaaGccaGLhWoaaiaawIcacaGLPaaaaeaacqWGqbaudaqadaqaaiabgYkaylabd+eapjabdchaWjabdwgaLjabdkhaYjabd+gaVjabd6gaUnaaBaaaleaacqWGPbqAcqWGQbGAaeqaaOWaaqqaaeaacuWG4baEgaWcamaaBaaaleaacqWGPbqAaeqaaOGaeiilaWIafmiEaGNbaSaadaWgaaWcbaGaemOAaOgabeaaaOGaay5bSdaacaGLOaGaayzkaaaaaiabg2da9maalaaabaGaemiuaa1aaeWaaeaacqWGpbWtcqWGWbaCcqWGLbqzcqWGYbGCcqWGVbWBcqWGUbGBdaWgaaWcbaGaemyAaKMaemOAaOgabeaaaOGaayjkaiaawMcaaaqaaiabdcfaqnaabmaabaGaeyiRaGTaem4ta8KaemiCaaNaemyzauMaemOCaiNaem4Ba8MaemOBa42aaSbaaSqaaiabdMgaPjabdQgaQbqabaaakiaawIcacaGLPaaaaaGaeyyXIC9aaSaaaeaacqWGMbGzdaqadaqaaiqbdIha4zaalaWaaSbaaSqaaiabdMgaPbqabaGccqGGSaalcuWG4baEgaWcamaaBaaaleaacqWGQbGAaeqaaOWaaqqaaeaacqWGpbWtcqWGWbaCcqWGLbqzcqWGYbGCcqWGVbWBcqWGUbGBdaWgaaWcbaGaemyAaKMaemOAaOgabeaaaOGaay5bSdaacaGLOaGaayzkaaaabaGaemOzay2aaeWaaeaacuWG4baEgaWcamaaBaaaleaacqWGPbqAaeqaaaGccaGLOaGaayzkaaGaeyyXICTaemOzay2aaeWaaeaacuWG4baEgaWcamaaBaaaleaacqWGQbGAaeqaaaGccaGLOaGaayzkaaaaaiaaxMaacaWLjaWaaeWaaeaacqaIYaGmcqaIWaamaiaawIcacaGLPaaaaaa@A61B@

where *P*(*Operon*_*ij*_) is the prior probability and the formula for the ratio on the right was given in Eq. 14. Given the individual pair probabilities and the mixture of four cases discussed above, the weight for each case is just its probability. For example, the weight for the three-gene operon case is

P(Operonijk|x→i,x→j,x→k)=P(Operonij|x→i,x→j)⋅P(Operonjk|x→j,x→k)     (21)
 MathType@MTEF@5@5@+=feaafiart1ev1aaatCvAUfKttLearuWrP9MDH5MBPbIqV92AaeXatLxBI9gBaebbnrfifHhDYfgasaacH8akY=wiFfYdH8Gipec8Eeeu0xXdbba9frFj0=OqFfea0dXdd9vqai=hGuQ8kuc9pgc9s8qqaq=dirpe0xb9q8qiLsFr0=vr0=vr0dc8meaabaqaciaacaGaaeqabaqabeGadaaakeaafaqabeGacaaabaGaemiuaa1aaeWaaeaacqWGpbWtcqWGWbaCcqWGLbqzcqWGYbGCcqWGVbWBcqWGUbGBdaWgaaWcbaGaemyAaKMaemOAaOMaem4AaSgabeaakmaaeeaabaGafmiEaGNbaSaadaWgaaWcbaGaemyAaKgabeaakiabcYcaSiqbdIha4zaalaWaaSbaaSqaaiabdQgaQbqabaGccqGGSaalcuWG4baEgaWcamaaBaaaleaacqWGRbWAaeqaaaGccaGLhWoaaiaawIcacaGLPaaaaeaaaeaacqGH9aqpcqWGqbaudaqadaqaaiabd+eapjabdchaWjabdwgaLjabdkhaYjabd+gaVjabd6gaUnaaBaaaleaacqWGPbqAcqWGQbGAaeqaaOWaaqqaaeaacuWG4baEgaWcamaaBaaaleaacqWGPbqAaeqaaOGaeiilaWIafmiEaGNbaSaadaWgaaWcbaGaemOAaOgabeaaaOGaay5bSdaacaGLOaGaayzkaaGaeyyXICTaemiuaa1aaeWaaeaacqWGpbWtcqWGWbaCcqWGLbqzcqWGYbGCcqWGVbWBcqWGUbGBdaWgaaWcbaGaemOAaOMaem4AaSgabeaakmaaeeaabaGafmiEaGNbaSaadaWgaaWcbaGaemOAaOgabeaakiabcYcaSiqbdIha4zaalaWaaSbaaSqaaiabdUgaRbqabaaakiaawEa7aaGaayjkaiaawMcaaaqaaiaaxMaacaWLjaWaaeWaaeaacqaIYaGmcqaIXaqmaiaawIcacaGLPaaaaaaaaa@7D60@

## Results

We tested OpWise on four data sets collected with a variety of measurement platforms (both glass sides and Affymetrix chips) that used different methods of controlling systematic bias (multiple probes per gene or dye swap) and from several different bacteria: With these data sets, we first used simulations to test whether OpWise fit the data and whether OpWise was robust to deviations from its assumptions. We then tested for systematic bias in the real data and examined significance estimates from OpWise and other methods. Finally, we tested whether operon-wise tests were more powerful than single-gene tests.

### Data sets

• dvSalt30 – *Desulfovibrio vulgaris *salt shock at 30 minutes (Z. He and J. Zhou, personal communication). This data was collected using two-color glass slides with 70-mer probes. The experiment was an indirect comparison through a genomic control. There were three biological replications for each condition, measured with one slide each, and two spots per gene per slide, for a total of six replicate measurements for each gene and condition.

• ecox – A comparison of aerobic and anaerobic log-phase growth in *Escherichia coli *(GEO accession GDS680, [19]). This data was from Affymetrix oligonucleotide chips with three or four replicate hybridizations for each of the two conditions.

• shCold5 – *Shewanella oneidensis *cold shock at 5 minutes (Z. He and J. Zhou, submitted). This data was a direct comparison of two-color glass slides using cDNA probes. There were five biological replicates with one slide each and two spots per gene per slide (10 measurements per gene total), but no dye swap (the same dyes were used for the control and treatment samples throughout).

• shHeat5 – *Shewanella oneidensis *heat shock at 5 minutes [20]. This data was also a direct comparison of two-color cDNA probes. There were three biological replicates, with two replicate slides each and two spots per gene per slide (12 total measurements per gene), and with dye swap (Cy3 dye was used for the treatment in half of the slides and for the control in the other half of the slides).

For the two-color direct comparison data sets (shCold5 and shHeat5), we performed intensity-dependent and then spatial normalization on each slide. Specifically, we first used a locally smooth estimator to remove intensity-dependent effects and then subtracted the median from each sector, similar to the recommendations of [6]. For the indirect comparison data set (dvSalt30), we treated the ratio of intensities between the channels corresponding to cDNA and to genomic DNA as a raw expression level. We first performed a global normalization for each slide so that the total expression level was the same for each slide, and then computed the average of the log-expression levels across slides from the two conditions. We then applied the intensity-dependent and spatial normalization approaches to these log-levels. For all three of these data sets, we considered the different spots for each gene as independent sets of replicates. There was little difference between within-slide and between-slide variance (data not shown). For the Affymetrix data set (ecox), the data we downloaded had already been normalized with dChip [21], so we used the normalized expression levels provided; to prevent small values of expression level from giving extreme outliers for log ratios, we added a small constant (5) to the expression levels before taking a logarithm.

For each data set, we also performed 50 simulations using the parameters estimated for that data set by OpWise. Each simulation had the same proportion of missing data as the corresponding data set. For operons, we randomly assigned adjacent genes on the same strand to be in the same operon or not with the probabilities given by the prediction method, but only if the probability was 0.5 or greater. With these simulations of the OpWise model, we were able to test our assumptions about the distribution of means and variances. To emulate the heavy tails in ecox (see below), we performed 50 simulations where 10% of the genes had much higher variation in the mean (a much lower *β*) than the other genes. Finally, to test our assumptions that (i) the true mean and true variance are correlated and (ii) the true variance is correlated within each operon, for each data set we performed 50 "uncoupled" simulations where the mean was independent of variance (the mean was normal with a fixed width) and genes in the same operon had independent variances.

### Fit of model to data

To see how well the model fit the data, we inferred the hyperparameters for each data set, used these parameters to create simulated data, and compared the simulated data to the original data sets. The model's inverse chi-square distribution gave an excellent fit to the observed distribution of squared deviance si2
 MathType@MTEF@5@5@+=feaafiart1ev1aaatCvAUfKttLearuWrP9MDH5MBPbIqV92AaeXatLxBI9gBaebbnrfifHhDYfgasaacH8akY=wiFfYdH8Gipec8Eeeu0xXdbba9frFj0=OqFfea0dXdd9vqai=hGuQ8kuc9pgc9s8qqaq=dirpe0xb9q8qiLsFr0=vr0=vr0dc8meaabaqaciaacaGaaeqabaqabeGadaaakeaacqWGZbWCdaqhaaWcbaGaemyAaKgabaGaeGOmaidaaaaa@3095@ [see Additional file 1]. The simulated distribution of observed means had heavier tails than a normal distribution, due to the wide spread of deviances. The distribution of means fit the data fairly well for three of the data sets, but for the ecox data set, the true distribution had even heavier tails [see Additional file 1].

To test our assumption that the variation in the true means depends on the true variances, we compared the correlations of observed means and squared deviances in the real data to simulations using the OpWise model and also using an uncoupled model in which the means and variances were independent. The observed mean and squared deviance were much more correlated than in the uncoupled model, except in the shCold5 data set [see Additional file 2]. Similarly, within each operon the squared deviances were significantly correlated [see Additional file 2]. However, the correlations were generally weaker than in the simulations, indicating deviations from the assumptions.

### Robustness of OpWise in simulations

To test OpWise, we created simulated data sets based on our statistical model. We wanted to verify that the estimated hyperparameters were accurate enough to give reasonable *p*-values. Because OpWise uses operons to estimate the overall reliability of the measurements, we also hypothesized that OpWise would be robust to the modest deviations from its assumptions. In particular, OpWise assumes that the variance in the true change of each gene depends on the variance of measurement for that gene. Because we found a weaker-than-expected relationship between observed deviances and means, we performed "uncoupled" simulations where the true means and variances were uncorrelated. Our statistical model also uses normal distributions. Although different genes can have widely varying variances of measurements, which allows the observed means to have somewhat heavy tails, even heavier tails were observed for the ecox data set. So, we also conducted heavy-tailed simulations (see Methods).

We examined the single-gene estimates of *P*(*μ*_*i *_> 0) for the simulated data (*μ*_*i *_is the true log-change for gene *i*). For the simulations using the OpWise model, we compared these *p*-values computed with estimated hyperparameters to "ideal" *p*-values computed with the true hyperparameters. For the "uncoupled" simulations with *μ*_*i *_independent of *σ*_*i*_, and for the heavy-tailed simulations, we compared the *p*-values to the actual sign of *μ*_*i *_for each gene.

**Figure 1:**
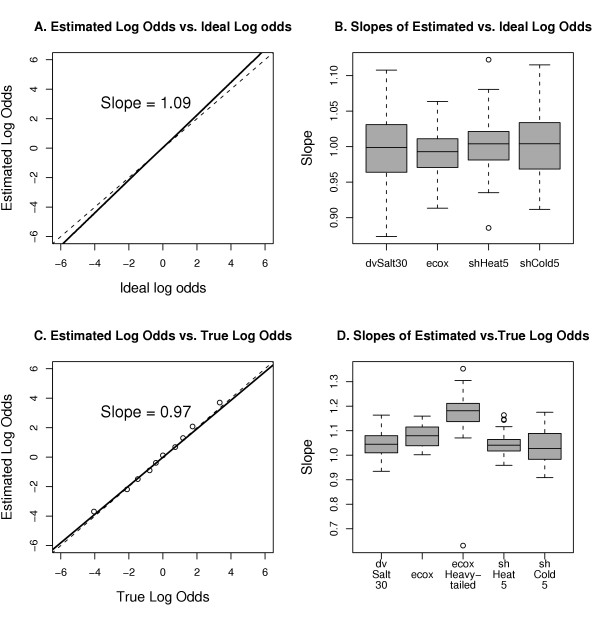
**Accuracy of *p*-values in simulations**. (A) A typical simulation matching the OpWise model. The solid line shows the estimated log odds for each gene (log⁡P(μi>0)1−P(μi>0)
 MathType@MTEF@5@5@+=feaafiart1ev1aaatCvAUfKttLearuWrP9MDH5MBPbIqV92AaeXatLxBI9gBaebbnrfifHhDYfgasaacH8akY=wiFfYdH8Gipec8Eeeu0xXdbba9frFj0=OqFfea0dXdd9vqai=hGuQ8kuc9pgc9s8qqaq=dirpe0xb9q8qiLsFr0=vr0=vr0dc8meaabaqaciaacaGaaeqabaqabeGadaaakeaacyGGSbaBcqGGVbWBcqGGNbWzdaWcaaqaaiabdcfaqnaabmaabaacciGae8hVd02aaSbaaSqaamXvP5wqSXMqHnxAJn0BKvguHDwzZbqegyvzYrwyUfgaiqGacaGFPbaabeaakiabg6da+iabicdaWaGaayjkaiaawMcaaaqaaiabigdaXiabgkHiTiabdcfaqnaabmaabaGae8hVd02aaSbaaSqaaiaa+LgaaeqaaOGaeyOpa4JaeGimaadacaGLOaGaayzkaaaaaaaa@4C40@) as a function of the "ideal" log odds based on the true values of the hyperparameters. The slope is from linear regression with the intercept fixed at zero. (B) Slopes from 50 simulations for each data set's hyperparameters. The boxes show the first and third quartiles and the medians, the whiskers show the most extreme point within 1.5 times the inter-quartile range of the box, and the points indicate outliers. (C) A typical "uncoupled" simulation where means and variances were independent. We sorted the genes by their estimated log odds into 10 bins of equal size. For each bin, a point shows the true log odds (from the number of genes with *μ*_*i *_> 0 and *μ*_*i *_< 0) and the average of the estimated log odds. Logistic regression gave a slope of 0.97 (solid line). (D) Slopes from 50 uncoupled simulations for each data set and from 50 heavy-tailed simulations for the ecox data set. The dashed lines in (A) and (C) show *x *= *y*.

When comparing the log odds of the estimated *p*-values to the log odds of the ideal *p*-values, we consistently observed a strongly linear relationship, with correlation coefficients above 0.9999 (see Figure 1A; logodds (p) ≡ log⁡p1−p
 MathType@MTEF@5@5@+=feaafiart1ev1aaatCvAUfKttLearuWrP9MDH5MBPbIqV92AaeXatLxBI9gBaebbnrfifHhDYfgasaacH8akY=wiFfYdH8Gipec8Eeeu0xXdbba9frFj0=OqFfea0dXdd9vqai=hGuQ8kuc9pgc9s8qqaq=dirpe0xb9q8qiLsFr0=vr0=vr0dc8meaabaqaciaacaGaaeqabaqabeGadaaakeaacyGGSbaBcqGGVbWBcqGGNbWzdaWcaaqaaiabbchaWbqaaiabbgdaXiabgkHiTiabbchaWbaaaaa@357E@). In other words, the ordering and shape of the significance values was not affected, but the overall scale of significance could be. To summarize this linear relationship between the two sets of significance estimates, we used the slope of the ideal log odds as a function of the estimated log odds. As shown in Figure 1B, most simulations had slopes very close to the ideal value of 1.0. In a total of 200 simulations across 4 data sets, the most extreme aggressive slope was 1.12 (for shHeat5). This corresponds to reporting *P*(*μ *> 0) = 0.964 when the true *P *= 0.95.

For the uncoupled and heavy-tailed simulations, which violated the assumptions of our model, we did not have ideal *p*-values to compare to, so we instead used logistic regression (*glm *in R, ) to estimate the slope. Logistic regression identifies the multiplier for the estimated log odds that best fits the observed pattern of whether *μ *> 0 or not – see Figure 1C. As shown in Figure 1D, the accuracy of OpWise was not dramatically affected by uncoupling the mean from the variance. However, the heavy-tailed simulations for the ecox data set produced slopes around 1.2, with a maximum of 1.35. (There was also one simulation with a very low slope, but this was due to a few extreme and biologically implausible values of *μ*_*i *_that are not present in our genuine data sets.) A slope of 1.35, which corresponds to reporting *P *= 0.982 when the true *P *= 0.95, is not ideal, but as we will show, methods that do not account for systematic bias, including non-parametric methods, can perform dramatically worse.

For all simulations, we also compared the operon-wise *p*-values to either the ideal or true significance. These gave similar slopes as the single-gene *p*-values, but with consistently smaller deviations from 1.0 (data not shown). Overall, OpWise was largely insensitive to deviations from its assumptions.

### Presence of bias

OpWise identified large amounts of systematic bias, similar in magnitude to the true changes in gene levels and the replication error, in all four data sets (Table 1). Furthermore, the bias was statistically highly significant in all four data sets, as determined by a maximum likelihood ratio test (see Table 1).

One source of apparent bias might be correlation between the replicates. That is, if the replicate measurements are not truly independent and some of the replicates are correlated, then the noise in the average of the replicate measurements will be larger than expected. For example, the shHeat5 data set had a total of 12 measurements per gene (3 biological samples times two slides per sample with dyes reversed times two spots per gene on each slide). In this data set, the replicate measurements with the same dye assignment were more correlated than those with reversed dyes. To test the pattern of bias with fully independent replicates, we created two subsets of the data. First, we used only the first spot for each gene on the slides and a single biological replicate, leaving two replicates with different dye assignments. Second, we used only a single dye assignment and only the first spot per slide, leaving three replicates from different samples. In both cases, we still observed large amounts of bias (data not shown). We also verified that OpWise was not sensitive to correlations between replicates. We created an exact duplicate of each replicate, and this "doubled" data set gave significance values very similar to the original data set (results not shown).

We also considered the possibility that mRNA levels in shCold5 and shHeat5, which were measured only 5 minutes after the stress was applied, were far from steady-state and that some operons would have poor agreement because of differential mRNA decay. However, later time points from these same experiments showed similar amounts of bias (data not shown). Overall, these analyses confirmed that systematic bias is a major problem in real data sets. Next, we show that ignoring this bias can lead to overestimating the significance of individual genes.

### OpWise estimates significance correctly

**Figure 2:**
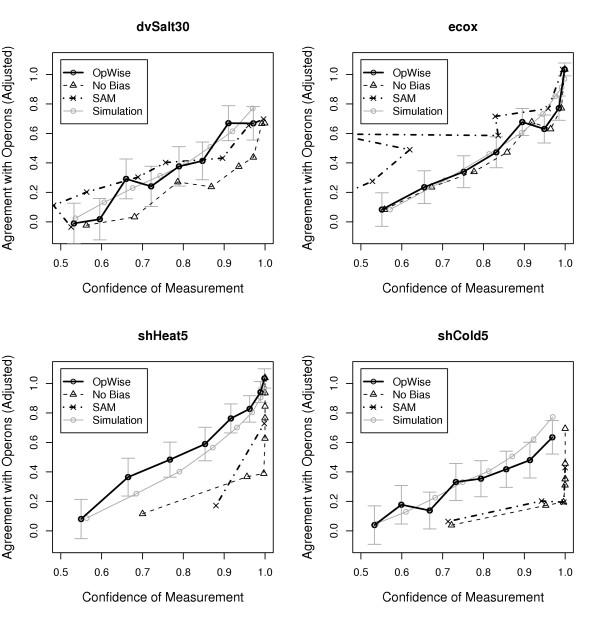
**Single-gene significance and agreement with operons**. For each data set and for three methods of assessing significance (OpWise, OpWise without bias, and significance analysis of microarrays), we divided the changers into eight groups of genes with different levels of confidence. The *x *axis shows the average confidence within each group of genes. For each group, the *y *axis shows the adjusted agreement with operon pairs (the adjusted proportion of pairs which have the same sign of log-ratio), which ranges from 0 for random data to 1 for perfect measurements. We also show average results from simulations for each data set (simulated and analyzed with the OpWise model). The error bars give the 95% confidence interval (from a *t *test) for the mean agreement for each group from the OpWise significance values. The odd left side of the ecox SAM curve is due to noise in the local FDR.

To test the quality of the significance estimates on real data, we compared the confidence assigned by OpWise to the extent of agreement with operons. Although our *p*-values are single-tailed – they test only the hypothesis that *μ*_*i *_> 0 – we wanted a two-tailed notion of confidence, because this is more comparable to other methods. We defined the two-tailed confidence as *C *= 2·|*p *- 1/2|. For each data set, we sorted genes by confidence into eight groups. For each gene, we then identified other genes predicted to be in the same operon, and asked whether the two genes changed in the same direction. (We used only adjacent genes, as operon predictions for non-adjacent genes are less confident.) Intuitively, if a group of genes are 99% confident changers, then 99% of the time, the measurement for that gene is correct, and it will always have the same sign as other genes in the operon; the other 1% of the time, there is no information about the gene, and the genes will have the same sign, by chance, 50% of the time. That is, *P*(*Agree*) = *C *+ (1 - *C*)/2, or 2·*P*(*Agree*) - 1 = *C *We also needed to correct for the possibility that the operon prediction is incorrect, which gives 2·*P*(*Agree*) - 1 = *C*·*P*(*Operon*). Thus, we defined an adjusted measure of agreement, whose expectation ranges from 0 for data that is all noise to 1 for perfect data, as *Adjusted *= (2·*Agree *- 1)/*P*(*Operon*), where *Agree *is 1 if true and 0 if false. This measure corrects for variations in the confidence of operon predictions between groups of genes – in some data sets, the most confident changers were, on average, in more confidently predicted operons (data not shown). Finally, even if the measurement for the first gene in the operon is highly confident and correct, the measurement for the other gene in the operon may be noisy, and the two genes may not agree. As there is no simple way to correct for this, we used the simulations described above, and compared the relationship between confidence and agreement in the real data to that in the simulations. The relationship between confidence and adjusted agreement with operons was approximately linear in all data sets (Figure 2) and was largely consistent with simulations [see Additional file 3].

Furthermore, for most groups of genes, including those with modest confidence values, the adjusted agreement with operons was much larger than zero. This suggests that the expression levels of all genes in these experiments were in fact changing, even if many individual genes could not be measured with confidence. In all four data sets, the top six of eight confidence groups had significantly more operon pairs that agreed with microarray data than not (all *p *< 0.05, binomial test). This confirmed our assumption that all genes are changers.

### Bias-free significance estimates are unreasonable

Figure 2 also shows the relationship between confidence and operons for our model without considering bias (using *γ *= ∞). Naturally, the confidence estimates from the model without bias were higher. In the shHeat5 and shCold5 data sets, the bias-free estimates of confidence were much too high: the highest and second-highest confidence groups both had confidence levels very near one, but the second-highest group had a much lower level of agreement with operons than the highest group. This also rules out one alternative explanation for why we detected significant bias in these data sets, which is that microarray data lacks bias but the operon predictions were flawed or systematically overconfident. In the latter case, the agreement with operons should have been lower for changers at every level of confidence, including the most confident changers. For dvSalt30, the bias-free confidence estimates appear to be more modestly over-confident, while for ecox, the difference between models with and without bias was small.

We also compared the confidence estimates from our model to those from a popular non-parametric method, SAM version 1.21 [4]. For each gene, SAM tests the null hypothesis that the gene's expression level is identical in the two conditions. SAM uses a modified *t *statistic with a pseudovariance term in the denominator, but rather than using a *t *test, SAM estimates the null distribution for the modified *t *statistic by performing random permutations of the data. SAM then uses the proportion of genes with high *p*-values to estimate the proportion of genes that are non-changers, and hence the proportion of genes that are true changers (similar to [7]). Finally, it corrects for multiple testing and estimates the false discovery rate (FDR). (For each gene, the FDR is an estimate of the proportion of false positives among genes that are at that gene's significance level or more significant.) To compare significance values from SAM to the confidence levels from OpWise in Figure 2, we needed the proportion of false positives within each group, also known as the local false discovery rate – the confidence is 1 minus the local FDR. For the most significant group, the local FDR is simply the FDR for the least significant member of the group. For the less significant groups, the number of false positives can be estimated from the FDR by subtracting the false positives expected for the more significant groups (similar to [22]).

As shown in Figure 2, for the shHeat5 and shCold5 data sets, SAM is far too confident, and is similar to the parametric model without bias. For the shHeat5 data set, SAM estimated an FDR of under 10^-4 ^for 2,284 genes, representing three quarters of all genes! In contrast, OpWise estimated that this group of genes was only 80% confident, implying a false discovery rate of 20%. The modest agreement with operons of these genes suggests that OpWise's estimate is reasonable (Figure 2). Indeed, the subset of the SAM significant changers that were *not *considered significant by the single-gene OpWise method (those with confidence < 0.95) showed much lower agreement with operons than those that were considered confident (83% vs. 97% of operon pairs changed in the same direction, *p *< 10^-13^, Fisher exact test). Reporting a FDR of 10^-4 ^when the true value is around 0.2 is far worse an overstatement of *p*-values than we ever observed in the OpWise simulations, even in those that violated our distributional assumptions (it would correspond to a slope of 6.6 in Figure 1D).

For the dvSalt30 data set, which has a moderate amount of bias, SAM was also more confident than our model, at least for the more significant changers (the three right-most groups containing the top 1,300 genes). The SAM curve was also noticeably below the simulation curve, suggesting that it was (moderately) over-confident. Finally, for ecox, which has little bias and a heavy-tailed distribution, SAM performed well (see top right of curve), while OpWise was perhaps slightly over-confident. Overall, we concluded that the bias OpWise inferred in these data sets was genuine, and that ignoring this bias (i.e., assuming that errors will average out over replicates) leads to unreasonable *p*-values.

### Operon-wise tests have greater power

**Figure 3:**
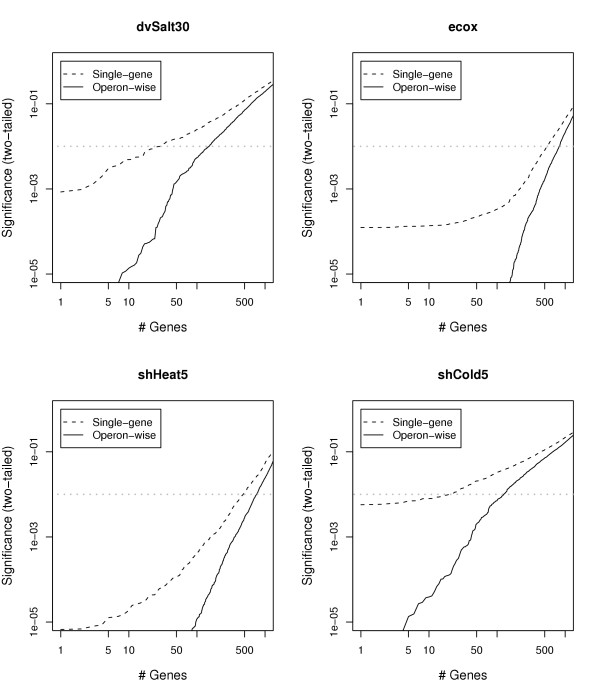
**Sensitivity of single-gene and operon-wise methods**. For each data set, we show the cumulative number of changers identified at varying levels of significance. Note the log scales. The horizontal line is at 0.01. Genes that are not in operons are included in the operon-wise results.

We hypothesized that when genes in operons have consistent measurements, higher confidence can be assigned to those measurements. We calculated "operon-wise" *p*-values that, for each gene, take into account the data for other genes in the same operon (if such genes exist; otherwise the operon-wise and single-gene *p*-values are identical). To test whether operon-wise *p*-values were more powerful than single-gene *p*-values, we compared the distributions of the operon-wise significance values to that of the single-gene significance values. Significance was defined as 1 - *C*. As shown in Figure 3, the operon-wise significance estimates are much more confident in each of the data sets, and at a significance cutoff of 0.01, 2–10 times more genes can be identified.

To summarize the performance of the various methods considered here – SAM, single-gene OpWise *p*-values, operon-wise *p*-values, and single-gene OpWise with bias ignored – we report the number of putative changers identified at a confidence threshold of 0.05 and the agreement with operons of those changers (Table 2). If bias is ignored, then single-gene OpWise generates similar results as SAM, but with bias accounted for, OpWise changers have much higher agreement with operons. This is probably because OpWise correctly identifies fewer genes as statistically reliable changers. The exception is the ecox data set, which has less bias (see Table 1), and hence all three methods give similar results. Compared to single-gene OpWise, the operon-wise method identifies more genes, which also show excellent agreement with operons, as this is part of how they are selected.

## Conclusion

We have described how operons can be used to detect systematic errors in measurements of prokary-otic gene expression patterns, to account for the bias when estimating significance, and to increase the confidence of measurements that are consistent within an operon. OpWise relies on the assumption that genes in the same operon have matching expression profiles. Although this assumption is only approximately correct, it is effective in practice, and is strongly preferable to ignoring the presence of systematic errors in the data. This assumption could be made more accurate by excluding from consideration those operon pairs that span an internal promoter or a partial terminator. Unfortunately, predicting alternative transcripts remains a challenging problem even in *E. coli *[23].

OpWise also relies on assumptions about the distributions of the true means and variances of the data. These assumptions are not entirely accurate, but without such assumptions, it would not be possible to distinguish low agreement within operons due to replication noise from that due to systematic bias. In simulations, OpWise was robust to the observed deviations from the assumptions.

In four data sets, OpWise identified significant and sometimes large amounts of systematic error. If this bias is not taken into account, as is generally the case with current approaches, then the statistical analysis can be far too aggressive. This bias is not an artefact arising from errors in operon predictions or from our distributional assumptions.

Likely sources for this bias include cross-hybridization or non-specific hybridization of some probes [10,21]. Indeed the data set without large amounts of bias (ecox) was collected using Affymetrix gene chips that use 15 probe sets per gene, and was normalized with a method that attempts to identify "bad" probes and remove them from the data [21].

Irrespective of bias and for all four data sets, the operon-wise method identified many more genes at any desired level of significance than the single-gene method. Although we only tested the operon-wise approach with one method for assessing significance, in principle, operon-wise *p*-values can be computed using single-gene *p*-values from any method. However, operon-wise *p*-values should not be used to rank genes, because consistent operons with modest changes can be ranked highly, and these could be indirect effects that are of low biological interest. Instead, we recommend setting a confidence threshold and then ranking all genes (or operons) above that confidence level by their fold-change. In any case, the main benefit of the present work is not for ranking or other broad exploratory analyses but in the ability to obtain reasonable *p*-values for specific hypotheses of the form "was gene X or operon Y upregulated in this experiment?" We also note that the benefit of OpWise is in assessing the reliability of the measurement, and not in estimating the amount of change for any gene.

As microarray technology becomes less expensive, experiment designs with high amounts of replication are becoming common. We observed that the systematic error can be comparable to or even larger than the variation between replicates. If systematic error is large relative to replication error, then performing many replicate measurements may not be cost-effective, and using several different probes for each gene might be preferable.

Finally, although the method we describe here requires operons and is only applicable to prokaryotic data, a similar approach might be useful for eukaryotes if there is prior knowledge of pairs of genes that have matching expression patterns. For example, stable complexes in yeast are often co-expressed [24], and the worm *C. elegans *has operons (but their co-expression may be weak [25]). In any case, our finding that statistical confidence levels from single probes can be misleading because of systematic bias probably applies to eukaryotic data.

## Availability and requirements

• **Project name: **OpWise

• **Project home page: **

• **Operating system(s): **Linux, Windows, MacOS

• **Programming language: **The R open-source statistics language 

• **Any restrictions to use by non-academics: **None

## Authors' contributions

M.N.P and E.J.A. conceived the project. M.N.P derived the method, analyzed the results, and wrote the manuscript. A.P.A. provided support and guidance. All authors edited the manuscript.

## Supplementary Material

Additional File 1 Distributions, in actual and simulated data, for observed means and squared total deviances Click here for file

Additional File 2 Relationship between means and variances in the data and in simulations Click here for file

Additional File 3 Single-gene significance and agreement with operons for additional simulations Click here for file

Additional File 4 OpWise.zip (includes source code in R, HTML instructions, and the data sets analyzed in this paper) Click here for file

## Figures and Tables

**Table 1:** Systematic bias in four biological data sets.

	dvSalt30	ecox	shHeat5	shCold5
Typical bias	0.25	0.12	0.37	0.88
Bias/signal (%)	70.4%	19.6%	49.9%	86.9%
Bias/replication error (%)	72.7%	35.8%	143.1%	199.1%
Bias/total (%)	52.4%	15.8%	47.2%	74.6%
Significance of bias				
Likelihood ratio	1.74e+02	9.38e+00	1.48e+03	1.81e+03
*p*-value	< 10^-77^	< 10^-5^	< 10^-646^	< 10^-786^

The typical size of the bias in the apparent log2-ratio is the square root of its variance, or E(1/θi⋅γ))
 MathType@MTEF@5@5@+=feaafiart1ev1aaatCvAUfKttLearuWrP9MDH5MBPbIqV92AaeXatLxBI9gBaebbnrfifHhDYfgasaacH8akY=wiFfYdH8Gipec8Eeeu0xXdbba9frFj0=OqFfea0dXdd9vqai=hGuQ8kuc9pgc9s8qqaq=dirpe0xb9q8qiLsFr0=vr0=vr0dc8meaabaqaciaacaGaaeqabaqabeGadaaakeaadaGcaaqaaiabdweafjabcIcaOiabigdaXiabc+caVGGaciab=H7aXnaaBaaaleaacqWGPbqAaeqaaOGaeyyXICTae83SdCMaeiykaKIaeiykaKcaleqaaaaa@3976@, where E(1/θi) = α/(ν - 1). The bias over the signal is the square root of the ratio of variances (β/γ
 MathType@MTEF@5@5@+=feaafiart1ev1aaatCvAUfKttLearuWrP9MDH5MBPbIqV92AaeXatLxBI9gBaebbnrfifHhDYfgasaacH8akY=wiFfYdH8Gipec8Eeeu0xXdbba9frFj0=OqFfea0dXdd9vqai=hGuQ8kuc9pgc9s8qqaq=dirpe0xb9q8qiLsFr0=vr0=vr0dc8meaabaqaciaacaGaaeqabaqabeGadaaakeaadaGcaaqaaGGaciab=j7aIjabc+caViab=n7aNbWcbeaaaaa@30F7@). The bias over the replicate error is also the square root of the ratio of variances (1/γ
 MathType@MTEF@5@5@+=feaafiart1ev1aqatCvAUfeBSjuyZL2yd9gzLbvyNv2CaerbwvMCKfMBHbqedmvETj2BSbqee0evGueE0jxyaibaieYdOi=BH8vipeYdI8qiW7rqqrFfpeea0xe9Lq=Jc9vqaqpepm0xbbG8FasPYRqj0=yi0lXdbba9pGe9qqFf0dXdHuk9fr=xfr=xfrpiWZqaaeaabiGaaiaacaqabeaabeqacmaaaOaa@2FC2@), and considers a single measurement (is not divided by the number of replicates). We also report the typical bias divided by the standard deviation of the observed log-changes mi. To show that the bias is statistically significant, we compared the likelihood ratio of the best-fitting model given systematic error to that without (with γ = ∞), using Eq. 10. Because we are testing whether γ lies at a boundary, in the absence of bias the distribution of 2·log(ratio) approximates a 50:50 mixture of two chi-squared distributions with 0 and 1 degrees of freedom [26].

**Table 2:** Genes with significant changes in expression as identified by OpWise methods and by SAM.

	**dvSalt30**		**ecox**		**shHeat5**		**shCold5**	
**Method**	**#Genes**	**%Agree**	**#Genes**	**%Agree**	**#Genes**	**%Agree**	**#Genes**	**%Agree**
1-gene (OpWise)	220	100%	1062	98%	1002	97%	187	100%
operon-wise	401	99%	1318	100%	1284	99%	374	100%
no-bias	1090	90%	1269	98%	3020	87%	3063	70%
SAM	852	94%	957	99%	3348	83%	3258	68%

For OpWise, genes were selected if the two-tailed confidence was 95% or higher (*P*(*μ*_*i *_> 0) < 0.025 or *P*(*μ*_*i *_< 0) > 0.975). For SAM, genes were selected if the false discovery rate was 5% or lower. For each method and for each data set, we report how many genes were selected as significant changers and what percentage of the operon pairs that contain those genes changed in the same direction. This "agreement" should be 100% for perfect microarray data and perfect operon predictions and 50% for random data.
