# Supplementary material for: OpWise: Operons aid the identification of differentially expressed genes in bacterial microarray experiments
Source: BMC Bioinformatics. 2006 Jan 13;7:19. doi: 10.1186/1471-2105-7-19 (PMC1397872; doi:10.1186/1471-2105-7-19)
Supplement: Additional File 1 — Distributions, in actual and simulated data, for observed means and squared total deviances [file 1471-2105-7-19-S1.pdf]

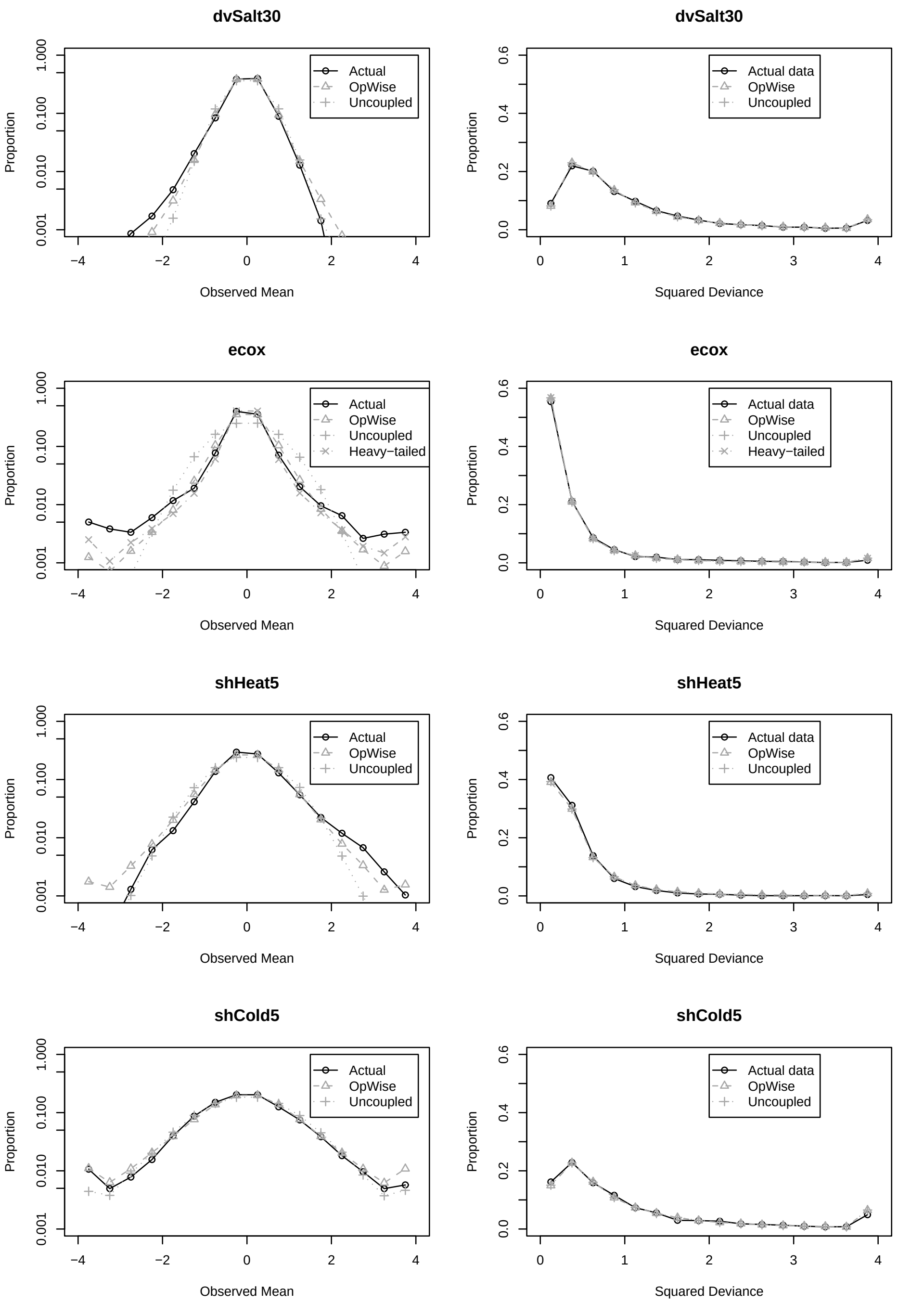

Additional File 1: Distributions, in actual and simulated data, for observed means (left) and squared total deviances (right). The leftmost and rightmost bins include all more extreme values. Note the log y-axis for the means.
