# Supplementary material for: OpWise: Operons aid the identification of differentially expressed genes in bacterial microarray experiments
Source: BMC Bioinformatics. 2006 Jan 13;7:19. doi: 10.1186/1471-2105-7-19 (PMC1397872; doi:10.1186/1471-2105-7-19)
Supplement: Additional File 2 — Relationship between means and variances in the data and in simulations [file 1471-2105-7-19-S2.pdf]

Additional File 2: Relationship between means and variances in the data and in simulations. r_S indicates a Spearman (ranked) correlation coefficient, and subscripts 1 and 2 indicate pairs of adjacent genes that are predicted to be in the same operon ($P(Operon) > 0.5$).

Data Set	$r_S(\|m\|, s^2)$	$r_S(m_1, m_2)$	$r_S(s_1^2, s_2^2)$
dvSalt30			
Actual data	0.209	0.405	0.137
OpWise	0.268	0.434	0.536
Uncoupled	0.123	0.469	-0.000
ecox			
Actual data	0.284	0.655	0.412
OpWise	0.339	0.708	0.554
Uncoupled	0.052	0.785	0.003
Heavy-tailed	0.313	0.545	0.547
shHeat5			
Actual data	0.235	0.727	0.169
OpWise	0.262	0.684	0.499
Uncoupled	0.065	0.684	0.002
shCold5			
Actual data	0.156	0.403	0.200
OpWise	0.328	0.465	0.573
Uncoupled	0.175	0.423	0.002
