# Supplementary material for: OpWise: Operons aid the identification of differentially expressed genes in bacterial microarray experiments
Source: BMC Bioinformatics. 2006 Jan 13;7:19. doi: 10.1186/1471-2105-7-19 (PMC1397872; doi:10.1186/1471-2105-7-19)
Supplement: Additional File 3 — Single-gene significance and agreement with operons for additional simulations [file 1471-2105-7-19-S3.pdf]

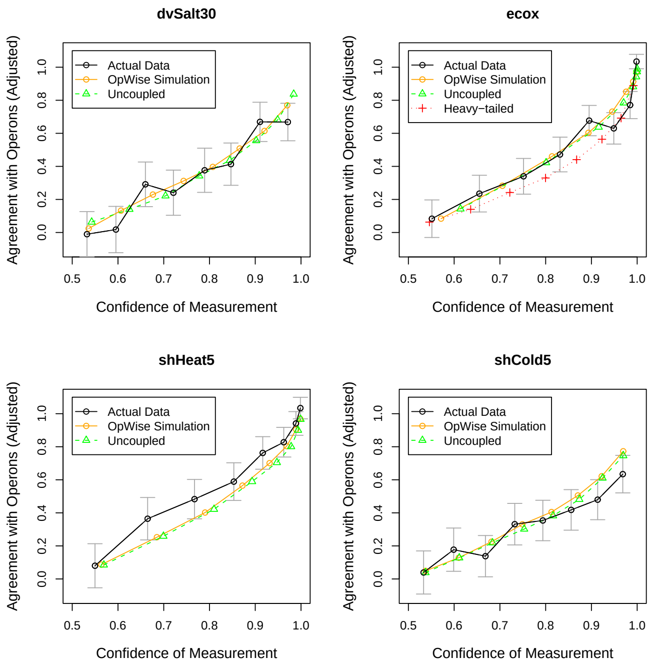

Additional File 3: Single-gene significance and agreement with operons. As in Figure 2, we divided the changers into eight groups of genes with different levels of confidence, and compare the average confidence within each group to the adjusted agreement with operon pairs. Here we compare the actual data set to additional "uncoupled" and (for ecox) heavy-tailed simulations. The means in the "uncoupled" simulations follow a normal distribution, while the real data sets and the other simulations have heavier tails.
