# Supplementary material for: OpWise: Operons aid the identification of differentially expressed genes in bacterial microarray experiments
Source: BMC Bioinformatics. 2006 Jan 13;7:19. doi: 10.1186/1471-2105-7-19 (PMC1397872; doi:10.1186/1471-2105-7-19)
Supplement: Additional File 4 — OpWise.zip (includes source code in R, HTML instructions, and the data sets analyzed in this paper) [file 1471-2105-7-19-S4.zip › OpWise/help.html]

Instructions on Using OpWise


|  |  |
| --- | --- |
|  | OpWise Instructions  System requirements OpWise is implemented in the open-source statistics programming language R, which runs on most platforms. The source code for OpWise is available here. OpWise typically takes between a few seconds and 2 minutes to analyze a microarray experiment. Data requirements OpWise requires two pieces of input: a microarray experiment with 2 or more replicates and operon predictions. The microarray experiment can be a direct comparison of two different samples on the same slide, or it can be an indirect comparison where both samples are compared to an external standard. OpWise does not do image processing -- it expects to receive log-ratios for each comparison. The data can be normalized already, or, OpWise can do intensity-based and sector-based normalization. For intensity-based normalization with a direct sample, the total intensity (the sum of the log of the two channels) can be supplied separately. The microarray data can easily be loaded into R from a tab-delimited file -- for example files, see the main OpWise page. OpWise uses a linear model, so extreme outliers (in either the mean or the variance) should be removed before processing, as should weak spots. They should be replaced with missing values (indicated with an empty field, and referred to in R as NA). Finally, OpWise does not do time series analyses -- in a time series, each time point must be analyzed separately (although they can all be in the same input file).  Operon predictions must be supplied as a list of pairs of adjacent genes on the same strand, and the probability of each pair being in the same operon. Operon predictions for most completely sequenced microbes are available from MicrobesOnline.  One practical problem is how to correspond the operon predictions from MicrobesOnline, which use VIMSS identifiers for the genes, to the identifiers in the microarray data. Usually the microarray data will include systematic identifiers of some sort, such as b3702 for E. coli K12, also known as VIMSS 17761 and better known as dnaA. The mapping between systematic identifiers and VIMSS ids is available from the operon predictions page for each organism, e.g., gnc83333.genes for E. coli. We will refer to this third piece of information as the mapping file. Running OpWise The first step is to load the source code and the input data files, e.g.:   ``` $ R > source("OpWise.R"); > dvSalt30Data <- read.delim("dvSalt30"); > mapping <- read.delim("gnc882.genes"); # not required for dvSalt30 which is already mapped > dvOperons <- read.delim("dvPred"); ```   The next step is to map the identifiers in the microarray data file to the VIMSS identifiers in the operon predictions. This step is not required for the dvSalt30 example, as this file already uses VIMSS ids. A typical call would be   ``` > mapped <- MapUarrayGenes(microarrayData, mapping, from="sysName", to="locusId"); ```   where mapping includes both sysName and locusId fields and microarrayData includes only sysName fields. The returned result (mapped) would then be the input to SummarizeUarrayVsOperons(). Names which do not map are given fake (negative) VIMSS ids, and will still be used for the analysis (but won't be predicted to be in operons). You can use the optional name argument to SummarizeUarrayVsOperons() (e.g., name=mapped$sysName) so that the statistics report from AnalyzeUarrayVsOperons() will include the original identifiers as well  Finally, to run the analysis, use SummarizeUarrayVsOperons() and AnalyzeUarrayVsOperons(), e.g.:   ``` > dvSalt30 <- SummarizeUarrayVsOperons(dvOperons, # the operon predictions 	dvSalt30Data$locusId,                     # the gene identifiers 	dvSalt30Data[,5:7],                       # the treatment replicates	 	dvSalt30Data[,8:10],                      # the control replicates (if an indirect comparison) 	spots=TRUE,                               # more than 1 spot per gene 	sweep=TRUE,                               # subtract the mean from each column (a normalization step) 	loess=TRUE,				  # do intensity-dependent normalization 	# do sector-based normalization, using row x column to define each sector         sector=interaction(dvSalt30Data$metaRow,dvSalt30Data$metaCol) ); > dvSalt30 <- AnalyzeUarrayVsOperons(dvSalt30,fold=1.5,base=2)    # Actually do the analysis ```   To do loess normalization for direct comparison data requires that the sum of the log intensities for each gene or spot be supplied separately, using the optional parameter A, for example with "A=log(data$I1)+log(data$I2)".  The single-gene p-values for each gene will be in dvSalt30$stats$p0, and the operon-wise p-values for each gene will be in dvSalt30$stats$pow0. These report the posterior probability that the true change for the gene is greater than zero. If you wish to use a confidence threshold of 5%, then values above 0.975 would be significant up-changers and values below 0.025 would be significant down-changers. Values near 0.5 indicate genes with little evidence for a change either way.  The analysis also reports the confidence that the gene changed by more than the fold-change specified by the fold parameter and in the measured direction (assuming that a logarithm, usually base 2, was applied to the data). These values are in dvSalt30$stats$pFold (for single-gene values) and dvSalt30$stats$powFold (for operon-wise values).  Finally, the analysis reports the normalized log-ratio, the shrunk estimate of the actual log-ratio, and a 95% confidence interval, in stats$muObs, stats$xShrunk, stats$xLo, and stats$xHi.  To export these values as a tab-delimited file (e.g. for viewing in Excel), use   ``` > write.table(dvSalt30$stats, "dvSalt30Stats", sep="\t", quote=FALSE, row.names=FALSE); ```    To see if the confidence values look reasonable, as in Figure 3 in the preprint, use   ``` > OperonAgreementVsConfidencePlot(dvSalt30) ```   The *y* axis will be the adjusted agreement with operons, and the *x* axis will be the confidence of measurement. This should give a roughly straight line.  To see the number of changers from single-gene and operon-wise p-values, as in Figure 4 in the preprint, use   ``` > SignificantChangersPlot(dvSalt30,bar=0.01); ```   where the optional bar parameter controls where the horizontal line is shown.  More examples To analyze E. coli aerobic vs. aerobic growth, 30 minutes (Covert et al. 2004>, use   ``` > ecPred <- read.delim("ecPred") > ecoxData <- read.delim("ecox"); > ecox <- AnalyzeUarrayVsOperons(SummarizeUarrayVsOperons(ecPred, 				ecoxData$VIMSS, 				log(5+ecoxData[,c("GSM18261","GSM18262","GSM18263")])/log(2), 				log(5+ecoxData[,c("GSM18286","GSM18287","GSM18288","GSM18289")])/log(2), 				sweep=FALSE, loess=FALSE, spots=FALSE)); ```   (ecox has already be normalized, but not log-transformed; adding a small constant prevents outliers from weak spots).  To analyze Shewanella oneidensis cold shock, 5 minutes (Jinzhong Zhou's group, VIMSS)), use   ``` > shPred <- read.delim("shPred") > shCold5Data <- read.delim("shCold5"); > shCold5 <- AnalyzeUarrayVsOperons(SummarizeUarrayVsOperons(shPred, 				shCold5Data$locusId, 				shCold5Data[,2:length(shCold5Data)], 				sweep=FALSE, loess=FALSE, spots=FALSE)); ```   (shCold5 has already been normalized and log-transformed, and is a direct comparison.)  To analyze S. oneidensis heat shock, 5 minutes (Gao et al. 2004), use  ``` > shPred <- read.delim("shPred") > shHeat5Data <- read.delim("shHeat5"); > shHeat5 <- AnalyzeUarrayVsOperons(SummarizeUarrayVsOperons(shPred, 					shHeat5Data$locusId, 					shHeat5Data[,2:13], 					sweep=FALSE, loess=FALSE, spots=FALSE)); ```   (shHeat5 has already been normalized and log-transformed, and is a direct comparison.) Further information  - A description of the method - Main OpWise page - Contact Eric Alm  ---  By Morgan N. Price, Adam P. Arkin, and Eric J. Alm |
