# Supplementary material for: OpWise: Operons aid the identification of differentially expressed genes in bacterial microarray experiments
Source: BMC Bioinformatics. 2006 Jan 13;7:19. doi: 10.1186/1471-2105-7-19 (PMC1397872; doi:10.1186/1471-2105-7-19)
Supplement: Additional File 4 — OpWise.zip (includes source code in R, HTML instructions, and the data sets analyzed in this paper) [file 1471-2105-7-19-S4.zip › OpWise/index.html]

OpWise -- Operon-wise analysis of microarrays


|  |  |
| --- | --- |
|  | OpWise  To estimate the reliability of bacterial microarray experiments, OpWise uses the agreement of measurements within operons to estimate the amount of systematic bias in the data. OpWise relies on the MicrobesOnline operons predictions. Using OpWise:  - Source code in R- Instructions- Sample data sets:       - Desulfovibrio vulgaris salt shock, 30 minutes (Jinzhong Zhou's group, VIMSS); not yet available)- E. coli aerobic vs. aerobic growth, 30 minutes (Covert et al. 2004)- Shewanella oneidensis cold shock, 5 minutes (Jinzhong Zhou's group, VIMSS))- S. oneidensis heat shock, 5 minutes (Gao et al. 2004)- Sample operon predictions:         - For D. vulgaris- For S. oneidensis- For E. coli  Further information:  - A description of the method - Contact Eric Alm  ---  By Morgan N. Price, Adam P. Arkin, and Eric J. Alm |
